# Global Epigenetic Changes Induced by SWI2/SNF2 Inhibitors Characterize Neomycin-Resistant Mammalian Cells

**DOI:** 10.1371/journal.pone.0049822

**Published:** 2012-11-28

**Authors:** Popy Dutta, Goutam Kumar Tanti, Soni Sharma, Shyamal K. Goswami, Sneha Sudha Komath, Marty W. Mayo, Joel W. Hockensmith, Rohini Muthuswami

**Affiliations:** 1 School of Life Sciences, Jawaharlal Nehru University, New Delhi, Delhi, India; 2 Department of Biochemistry and Molecular Genetics, University of Virginia, Charlottesville, Virginia, United States of America; Florida State University, United States of America

## Abstract

**Background:**

Previously, we showed that aminoglycoside phosphotransferases catalyze the formation of a specific inhibitor of the SWI2/SNF2 proteins. Aminoglycoside phosphotransferases, for example neomycin-resistant genes, are used extensively as selection markers in mammalian transfections as well as in transgenic studies. However, introduction of the neomycin-resistant gene is fraught with variability in gene expression. We hypothesized that the introduction of neomycin-resistant genes into mammalian cells results in inactivation of SWI2/SNF2 proteins thereby leading to global epigenetic changes.

**Methodology:**

Using fluorescence spectroscopy we have shown that the inhibitor, known as Active DNA-dependent ATPase A Domain inhibitor (ADAADi), binds to the SWI2/SNF2 proteins in the absence as well as presence of ATP and DNA. This binding occurs via a specific region known as Motif Ia leading to a conformational change in the SWI2/SNF2 proteins that precludes ATP hydrolysis. ADAADi is produced from a plethora of aminoglycosides including G418 and Streptomycin, two commonly used antibiotics in mammalian cell cultures. Mammalian cells are sensitive to ADAADi; however, cells stably transfected with neomycin-resistant genes are refractory to ADAADi. In resistant cells, endogenous SWI2/SNF2 proteins are inactivated which results in altered histone modifications. Microarray data shows that the changes in the epigenome are reflected in altered gene expression. The microarray data was validated using real-time PCR. Finally, we show that the epigenetic changes are quantized.

**Significance:**

The use of neomycin-resistant genes revolutionized mammalian transfections even though questions linger about efficacy. In this study, we have demonstrated that selection of neomycin-resistant cells results in survival of only those cells that have undergone epigenetic changes, and therefore, data obtained using these resistant genes as selection markers need to be cautiously evaluated.

## Introduction

The SWI2/SNF2 proteins play an important role in maintaining the cellular epigenome by harnessing the energy released by ATP to mobilize nucleosomes, and thus remodel chromatin architecture [1]–[3]. Consequently, inhibition of the SWI2/SNF2 proteins could alter the epigenome and we herein explore the effects of one such inhibitor derived from aminoglycosides.

Aminoglycosides, including neomycin and G418, are a large family of bactericidal antibiotics that interact with nucleotide A1408 present in the A-site of 16S rRNA [4], [5], thus inhibiting translocation. Aminoglycosides are also toxic to eukaryotic cells, however the mechanism of action is unclear, as the eukaryotic rRNA contains a guanine residue in lieu of adenine at position 1408 thus precluding aminoglycoside binding [4], [6].

Prokaryotic resistance to aminoglycosides is mediated by many means, one of which includes a reaction catalyzed by prokaryotic APH, therein transfering a phosphate group from ATP to the 3′ position of aminoglycosides to generate a phosphoaminoglycoside as the predominant product [7]–[9]. Mammalian cells transfected with *aph* acquire resistance to aminoglycosides and therefore, the gene has been used extensively as a selection marker [10] wherein transfected cells are selected using neomycin or G418.

We have identified a hitherto uncharacterized product of APH enzymatic action which we call ADAADi [11]. ADAADi specifically inhibits Active DNA-dependent ATPase A Domain (ADAAD) and other members of the SWI2/SNF2 protein family [11]. This has been confirmed by Felle et al. who showed that this product inhibits nucleosome translocation and thereby prevents translocation of RNA polymerase I on chromatin templates [12]. Their experiments further confirmed that the molecule is a potent inhibitor of Snf2, ISWI, and CHD1 proteins while having no effect on other ATPases [12].

The identification of the inhibitor led us to ask what happens when mammalian cells are stably transfected with APH. Given the essentiality of SWI2/SNF2 proteins in eukaryotic cells, we hypothesized that G418 resistant *aph* transfected cells would also be ADAADi resistant. Further, the selection pressure on the transfected cells to survive in the presence of inactive SWI2/SNF2 proteins would induce these cells to effect alterations of an epigenetic nature that would be manifested as altered gene expression. Such alteration would occur in the absence of the introduction of any gene products other than *aph*.

In this manuscript we have used a multipronged approach to understand the changes occurring in neomycin-resistant mammalian cells. Using biophysical techniques, we show that ADAADi binds specifically to a region known as Motif Ia in SWI2/SNF2 proteins and this interaction induces a conformation change in the protein that prevents ATP hydrolysis. We show ADAADi can be generated from a wide range of aminoglycosides including G418 and streptomycin. We then show that ADAADi is toxic to mammalian cells; however, as hypothesized, cells stably transfected with APH are resistant to ADAADi. This resistance is due to inactivation of endogenous SWI2/SNF2 protein resulting in changes in the epigenome and thus, alterations in gene expression patterns. From these results we conclude that mammalian cells transfected with *aph* and selected in the presence of G418 result in survival of only those cells that attain epigenetic alterations which likely account for the widely variable results often obtained with this heterologous selection system.

## Materials and Methods

### Chemicals

All chemicals were purchased from Merck, Qualigens, or Sigma-Aldrich unless specified otherwise. Radiolabeled [^32^P] γATP was purchased from Bhabha Atomic Research Center, Mumbai, India. RNAP II antibody was purchased from Cell Signaling Technology. The antibody against the HARP region of human SMARCAL1 was raised by Bangalore Genei (India). The list of primers used in RT-PCR and ChIP assays is given in Table S1.

### Purification of ADAAD

His-ADAAD, used for binding studies, was purified as described previously [13]. For mapping and CD studies, ADAAD as well as deletion constructs were expressed as GST-fusion protein in BL21 (DE3) cells and purified as described previously [14]. In case of deletion constructs the GST-tag was not cleaved as the resultant proteins were not stable.

### Cloning of aph (3′)-I, aph (3′)-IIa and aph (3′)-IIIa

The primers used for amplifying *aph (3*′*)-I* and *aph (3*′*)-IIa* is provided in Table S2. The PCR products were cloned into pET-14b vector. *aph (3*′*)-IIIa* was released from the parent vector pSACG1 by digesting with NdeI and XhoI and ligated into pET-14b vector.

### Purification of APH

The cells overexpressing APH were induced with 1 mM IPTG for 4 hours at 37°C. The cells were lyzed in buffer containing of 50 mM Tris.Cl (pH7 5), 200 mM NaCl, 1 mM PMSF, 0.2 mM β-mercaptoethanol, and 0.1 mg/ml lysozyme) and the protein was purified using Ni^+2^-NTA agarose column.

### Protein estimation

Protein was estimated using Bradford reagent. The absorbance was recorded at 595 nm using Spectramax microplate reader (MTX Lab Systems, Inc, USA).

### Synthesis and purification of inhibitor

ADAADi was synthesized and purified using Bio-Rex 70 anion exchanger as described previously [11]. The fractions were analyzed by thin layer chromatography (TLC) using Silica gel 60 plates (Merck) in methanol: ammonium hydroxide (5:2 v/v) solvent system. After desalting, the inhibitor was scrapped off from TLC plates and resuspended in 2 ml of Solution A (chloroform: methanol: water::20:40:1). The mixture was sonicated, vortexed, and centrifuged at 5000 rpm for 5 min. The supernatant, containing the inhibitor, was dried and dissolved in distilled water. The concentration of the inhibitor was estimated using neomycin as standard on TLC plate.

### Fluorescence measurements

Fluorescence was measured at 25°C using Cary-Varian spectrofluorimeter as described previously [13]. Stem-loop DNA (5′- GCGCAATTGCGCTCGACGATTTTTTAGCGCAATTGCGC-3′), synthesized by Sigma-Aldrich, was used in these studies. The intrinsic fluorescence quenching data obtained with acrylamide was analyzed using Stern-Volmer plots as described previously [13], [14].

### CD spectroscopy studies

Far UV CD spectra between 200–260 nm were obtained using 0.1 mg/ml protein as described previously [13], [14].

### ATPase assays

ATPase activity of purified ADAAD in the absence and presence of inhibitor was measured using NADH oxidation assay as described previously [11]. *In vivo* SMARCAL1 activity was assessed using NADH oxidation assay in untransfected and stably transfected Neuro2A cells by immunoprecipitating the protein.

### Immunoprecipitating SMARCAL1 for ATPase activity estimation

Cells were lysed by incubating at 4°C for 15 minutes in lysis buffer (50 mM Tris.Cl pH 7.5, 400 mM NaCl, 1 mM EDTA, 1 mM EGTA, 0.1% NP-40, 1 mM PMSF, and protease inhibitor cocktail). After sonication in water bath (10 s ON; 50 s OFF) for 4 minutes, the lysate was clarified by centrifuging at 13,000 rpm for 10 minutes at 4°C. The supernatant, after pre-clearing with protein-A beads, was incubated with polyclonal antibodies against SMARCAL1 overnight at 4°C, and the ATPase activity of the immunoprecipitated SMARCAL1 was estimated using NADH oxidation assay. In these experiments 5 μM ADAADiN, and 5 μM ADAADiG418 was used and % ATPase activity was estimated with respect to the ATPase activity in untransfected cells in the absence of ADAADi.

### Cell culture

Neuro2Acells were purchased from Cell repository, NCCS, Pune, India and the C2C12 mouse myoblast cell line (ATCC) and its derivatives were a gift from Dr. Cun-Yu Wang (University of Michigan) [15]. Neuro2Acells were maintained in DMEM containing 10% fetal bovine serum (FBS), and 1% penicillin-streptomycin-amphotericin cocktail. The C2C12 mouse myoblast cell line were maintained in DMEM with 4 mM L-glutamine adjusted to contain 1.5 g/L sodium bicarbonate and 4.5 g/L glucose, and 10% FBS.

### Creating stable Neuro2A transfectants

pcDNA 3.1 myc/his (-) vector as well as SG2NA constructs were linearized with ScaI, gel purified and transfected into Neuro2A cells using lipofectamine 2000 as per manufacturer's protocol (Life Technologies). 72 hours post-transfection the cells were trypsinized and transferred to 100 mm dish. The stable transfectants were selected in the presence of 400 μg/ml G418. Clones formed after 3 weeks were transferred to new plates and counted as passage 1. Cells were maintained in the presence of 100 μg/ml G418 unless otherwise stated.

### Cell viability assays

5000 cells were seeded in a total volume of 200 μl in a 96-well plate. The transfected cells were grown for 24 hours as explained in Figure S6. After 24 hours, the media was aspirated and fresh media containing pen-strep along with increasing concentration of either parent aminoglycoside or ADAADi was added. The fraction of cells surviving after 60 hours treatment with the parent aminoglycoside or ADAADi was estimated using MTT assay (Sigma-Aldrich).

### Real-time PCR

RNA was extracted using Tri reagent (Sigma-Aldrich). cDNA was generated using 3 μg of total RNA and semi-quantitative PCR reactions were performed using the Taq DNA polymerase and gene-specific primers for 20 cycles. Real-time PCR was done in 20 μl volume using 1X SYBR Green Master Mix (Applied Biosystems), 10 pmole of gene specific primers and 1 μl of cDNA to amplify transcripts. Reaction was performed in an Applied Biosystems 7500 Real- Time PCR System (50°C, 2 min; 95°C, 10 min, 1 cycle; 95°C, 15 s; 60°C 1 min, 40 cycles). β-actin or GAPDH was used as an internal control for normalization. The primers used for real-time PCR is provided in Table S1.

### Preparation of cell lysate for western blot

To analyze endogenous SG2NA levels, the cells were lysed in buffer containing 50 mM Tris.Cl pH 7.6, 400 mM NaCl, 1 mM EDTA, 1 mM EGTA, 1% NP-40, 1 mM sodium orthovanadate, 10 mM sodium fluoride, protease inhibitor cocktail and 1 mM PMSF. Ice-cold urea buffer (90% 8.8 M urea, 2% (v/v) 5 M sodium phosphate and 8% 1 M Tris.Cl pH 8.0) was used to lyse cells for analysis of SMARCAL1, Brg1, and Rad54.

The levels of modified histones were analyzed by boiling 3×10^4^ cells for 15 minutes in 10 μl of 6X protein loading dye and resolving on 12% SDS-PAGE for western blot analysis.

### Western blot

Cell lysates (100 μg) were resolved on 10% SDS-PAGE and transferred to PVDF membranes in Towbin's buffer (25 mM Tris, 192 mM glycine and 20% (v/v) methanol). Following transfer, the membranes were blocked at room temperature with 3 % (w/v) BSA in TBS containing 0.05% Tween-20 (TBST) and incubated overnight at 4°C with respective primary antibodies at appropriate dilutions. Subsequently, the blots were extensively washed with TBST, incubated for 45 min at room temperature with respective secondary antibody (at appropriate dilutions) and developed using Enhanced Chemiluminescence detection system (Sigma-Aldrich).

### Chromatin Immunoprecipitation assays

The cells were fixed with 1% formaldehyde and resuspended in buffer containing 10 mM Tris.Cl (pH 8.0), 140 mM NaCl, 1 mM EDTA, 1% Triton-X, 0.1% sodium deoxycholate, 10 mM sodium butyrate, 1 mM PMSF and protease inhibitor cocktail. DNA was fragmented by sonicating the cells for 30 min (60 cycles of 30 sec on and 30 sec off). The chromatin (200 µg in 500 μl reaction volume) after pre-clearing with protein A-agarose beads was incubated overnight at 4°C with 5 µg of appropriate antibody. The chromatin-antibody complex was immunoprecipitated with protein A-agarose beads, washed extensively, and eluted with 100 µl of elution buffer (containing 100 mM (NH_4_)_2_CO_3_ and 1% SDS). The cross-link was reversed using 10 μg/ml RNase A and proteinase K, DNA was extracted using phenol:chloroform and precipitated using 1/10^th^ volume of NaOAc and 2 volumes of ethanol. For control experiments IgG beads (Sigma-Aldrich) were used. The primers used for ChIP analysis is provided in Table S1.

### Microarray analysis

Microarray analysis was performed using Agilent platform by Genotypic Technology (India). The data is available at GEO website (GEO 36142).

### Immunocytochemistry

The cells were fixed with chilled methanol, blocked for 2 hours using 1% BSA in 1X PBS, and incubated with primary antibody for 1 hour. The cells were washed and incubated with secondary antibody for 1 hour before imaging using confocal microscope (Olympus).

## Results

### ADAADi , a distinct product generated by APH

**Figure 1:**
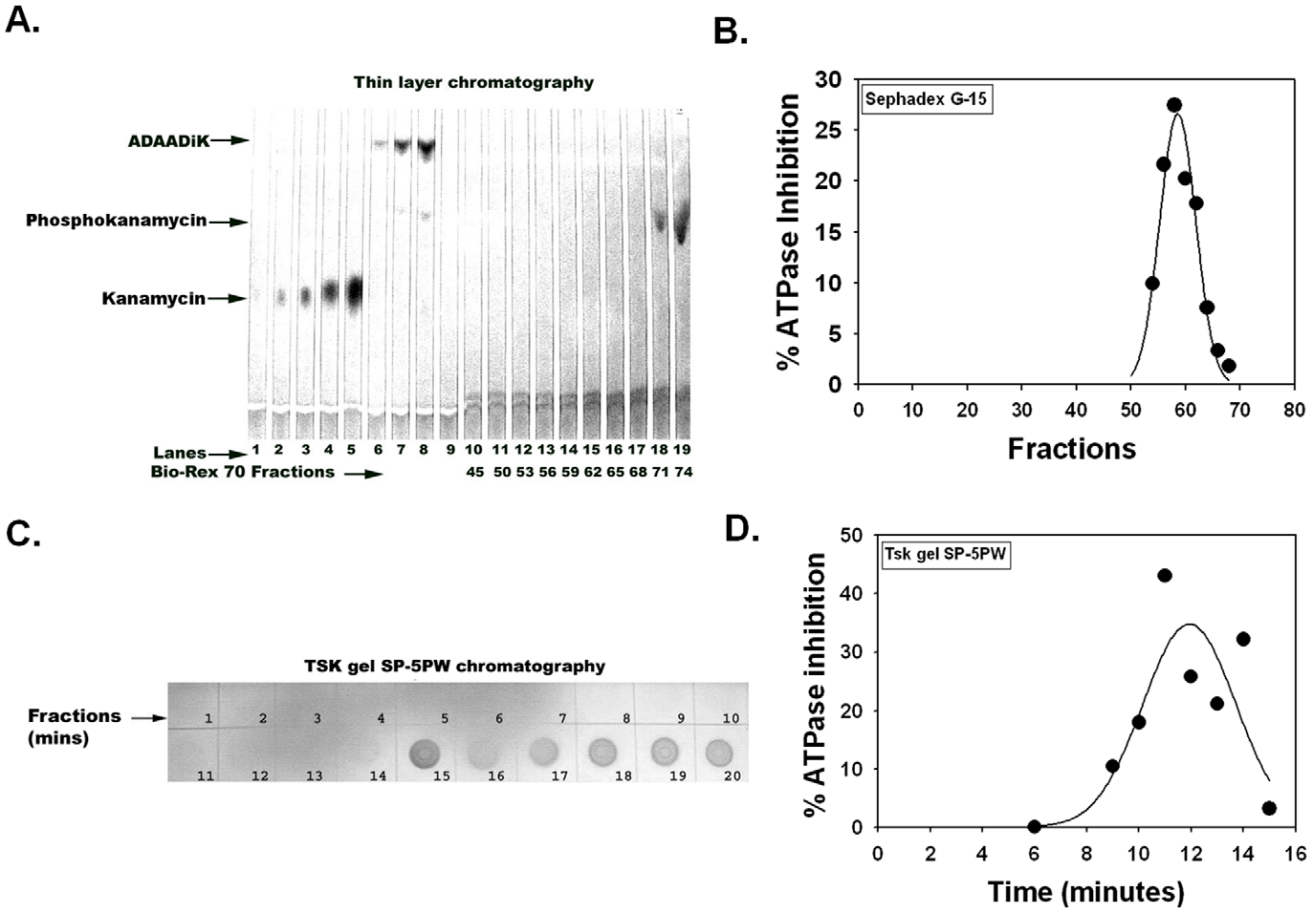
ADAADi is produced by APH. (A). Analysis of ADAADiK on silica 60A plate by TLC after purification using Bio-Rex 70 followed by G-15 desalting column. Kanamycin (lanes 1-6), ADAADiK (lanes 7–9), and phosphokanamycin (lanes 18–19) migrate with different mobilities and thus can be separated on these plates. (B). Inhibition profile of fractions eluted from G-15 column. (C). Purification of ADAAD using TSK gel SP-5PW column. The ninhydrin sensitive spots (fractions 15–20) correspond to phosphokanamycin and kanamycin, while the ADAADiK concentration (fractions 9–14) is too low to be detected by ninhydrin. (D). Inhibition profile of fractions eluted from SP column.

Previously we have shown APH (3′)-IIIa generates a product from aminoglycosides, kanamycin and neomycin, that inhibits the ATPase activity of the SWI2/SNF2 proteins [11]. This product, now named ADAADi, can be separated from the known 3′-phosphoaminoglycoside derivatives as well as the parental aminoglycoside using a Sephadex column (Figure 1A). Analysis of column fractions using TLC (lanes 10–19/fractions 45–74) showed a ninhydrin-sensitive spot corresponding to phosphokanamycin (fractions 71 and 74), while the concentration of ADAADiK was too low to be detected. However, upon enzymatic assay, the inhibitor ADAADiK was found exclusively in fractions 53–68 (Figure 1B). ADAADiK concentrated from multiple column runs can be visualized as a chromatographically-distinct ninhydrin-sensitive spot on a TLC plate, demonstrating a higher mobility than either kanamycin or phosphokanamycin (Figure 1A; lanes 6–8). ADAADiK (fractions 9–14) also elutes prior to phosphokanamycin and kanamycin (fractions 15–20) on a HPLC TSK gel SP-5PW column (Figure 1C and D). Acid hydrolysis of the peak fractions demonstrated ADAADiK as constituting <0.58% of the phosphorylated aminoglycoside product, which accounts for our need to concentrate the ADAADi in Figure 1 and the failure to historically identify this product using physical methods.

### Interaction of ADAADi with ADAAD

To understand the interaction of ADAADi with ADAAD, a member of the SWI2/SNF2 family, we synthesized ADAADi using kanamycin and neomycin. These derivatives, ADAADiK and ADAADiN respectively, were purified and titrated with protein while monitoring the quenching of the intrinsic tryptophan fluorescence. Analysis of the data demonstrated that both ADAADiN and ADAADiK bind to ADAAD in the absence of either ATP or stem-loop DNA (slDNA), a well-characterized DNA effector of ADAAD [13]. The data fit well to a one-site saturation model, suggesting that the ADAADi-ADAAD interaction was predominantly via a single site, and the K_d_ was calculated to be 35.8±5.0 nM for ADAADiN and 21.9±4.2 nM for ADAADiK (Figure S1A and S1B; Table 1). Further, binding data for ADAADiN-ADAAD interaction showed that the inhibitor was able to bind to ADAAD in the presence of 40 μM ATP as well as in the presence of 3 μM slDNA with similar binding constants (Figure S2A and S2B; Table 1). This binding constant was same as that in the absence of ATP and slDNA. A similar result was obtained with ADAADiK (Figure S3A and S3B; Table 1). We thus conclude that the interaction of the inhibitor with ADAAD is independent of these two ligands.

**Table 1:** Interaction of ADAADiN and ADAADiK with ADAAD in absence and presence of ATP and slDNA.

Condition	Ligand	K_d_ (M)	R^2^
ADAAD +	ADAADiN	(35.8±5.0)×10^−9^	0.97
	ADAADiK	(21.9±4.2)×10^−9^	0.97
	*ATP	(1.6±0.5)×10^−6^	0.98
	*slDNA	(19.9±4.9)×10^−9^	0.98
ADAAD + ATP +	ADAADiN	(18.1±4.3)×10^−9^	0.98
	ADAADiK	(20.4±2.0)×10^−9^	0.99
ADAAD + slDNA +	ADAADiN	(23.3±2.7)×10^−9^	0.99
	ADAADiK	(36.3±4.0)×10^−9^	0.99
ADAAD + ADAADiN +	ATP	(0.1±0.003)×10^−6^	0.99
	slDNA	(0.63±0.1)×10^−9^	0.97
ADAAD + ADAADiK +	ATP	(0.1±0.01)×10^−6^	0.99
	slDNA	(0.46±0.11)×10^−9^	0.97

* The values were reported in Nongkhlaw *et al*. (Nongkhlaw, 2009).

His-ADAAD was used for these studies.

Next, we investigated the interaction of ATP and slDNA in the presence of ADAADi. Previously, we have shown that ATP binds to ADAAD with a K_d_ of 1.6±0.5 μM while slDNA binds to ADAAD with a K_d_ of 19.9±4.9 nM (Table 1) [13]. Analysis of the binding data when ADAAD was saturated with 2 μM ADAADiN shows that both ATP and slDNA bind with higher affinity. Thus, ATP binds to ADAAD with a K_d_ of 0.1±0.03 μM while slDNA binds to ADAAD with a K_d_ of 0.63±0.1 nM when the protein is saturated with ADAADi (Figure S2C and S2D; Table 1).

A similar result was obtained when ADAAD was saturated with ADAADiK (Figure S3C and S3D; Table 1).

### Role of Motif Ia in ADAADi-ADAAD interaction

ADAAD contains the conserved helicase motifs (Table 2) [14]. Three deletion constructs of ADAAD-MAD47, MAD33, and MAD53-lacking DNA-dependent ATPase activity were used to delineate the interaction region (Table 2). The K_d_ values for the interaction of ADAADiN with MAD47 and MAD 33 were similar to that of wild type leading to the conclusion that motifs Q, I, and Ia are sufficient for the ADAADi-ADAAD interaction. In contrast, these three motifs are not sufficient for the slDNA-ADAAD interaction (Table 2). Further, the K_d_ for ADAADiN- MAD53 binding leads us to conclude that motif Ia may be important for inhibitor-ADAAD interaction but not for slDNA-ADAAD interaction which is distinct from the 18 but many otherulturesipulation of eukaryotic cells.ext postulated that the presence of (Table 2).

**Table 2:** Delineating the motifs required for the interaction of ADAADiN with ADAAD.

Protein	Motifs present	K_d_ (M) slDNA	R^2^	K_d_ (M) ADAADiN	R^2^
Wild type	Q, I, Ia, II, III, IV, V, VI	(3.8±1.1) ×10^−9^	0.99	(35.8±5.0) ×10^−9^	0.97
MAD 47	Q, I, Ia, II, III	(6.5±1.0) ×10^−9^	0.99	(18.5±5.4) ×10^−9^	0.94
MAD 33	Q, I, Ia	(41.5±1.5) ×10^−9^	0.99	(26.9±1.8) ×10^−9^	0.97
MAD 53	Ia, II, III, IV, V, VI	(8.2±1.5) ×10^−9^	0.98	(50.5±8.7) ×10^−9^	0.98
MAD28	Q, I	(22.1±5.1) ×10^−9^	0.97	ND	-
MAD8	Ia	(8.7±2.0) ×10^−9^	0.98	(31.9±7.2) ×10^−9^	0.97

ND  =  not determined.

ADAAD as well as the deletion constructs were expressed as GST fusion protein and purified using glutathione agarose beads. In case of ADAAD and AMD47, GST was cleaved using PreScission protease and the purified protein was used in these studies.

**Figure 2:**
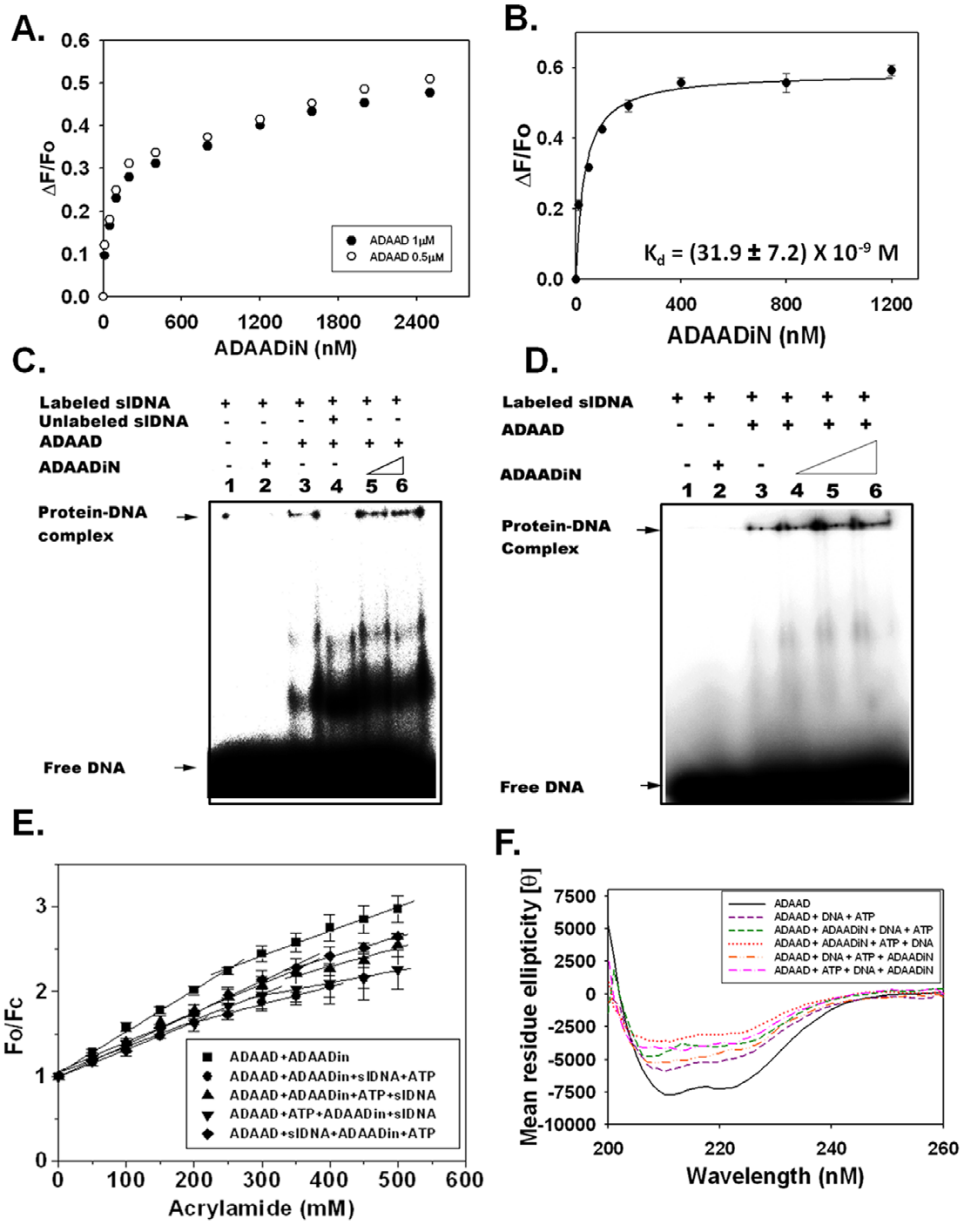
ADAADi binds to Motif Ia of ADAAD leading to conformational changes in the protein. (A). MAD28 (0.5 μM and 1.0 μM) was titrated with increasing concentration of ADAADiN. (B). MAD8 (0.5 μM) was titrated with increasing concentration of ADAADiN. (C). **Electrophoretic mobility shift assay**. Lane 1: Free slDNA; Lane 2: slDNA with 1 μM ADAADiN; Lane 3: slDNA with ADAAD; Lane 4: slDNA with ADAAD and 15 μM unlabeled slDNA; Lane 5: slDNA with ADAAD and 1 μM ADAADiN; Lane 6: slDNA with ADAAD and 2 μM ADAADiN. (D). Lane 1: Free slDNA; Lane 2: slDNA with 1 μM ADAADiN; Lane 3: slDNA with ADAAD; Lane 4: slDNA with ADAAD and 4 μM ADAADiN; Lane 5: slDNA with ADAAD and 8 μM ADAADiN; Lane 6: slDNA with ADAAD and 10 μM ADAADiN. (E). **Stern-Volmer plots**. ADAAD (0.5 μM) was titrated with increasing concentration of acrylamide in presence of either 2 μM ADAADiN (▪), or 2 μM ADAADiN, 3 μM slDNA and 40 μM ATP (•), or 2 μM ADAADiN, 40 μM ATP and 3 μM slDNA (▴), or 40 μM ATP, 2 μM ADAADiN and 3 μM slDNA (▾), or 3 μM slDNA, 2 μM ADAADiN, and 40 μM ATP (⧫). (F). The CD spectra of ADAAD in the absence and presence of ADAADiN (0.2 μM), ATP (20 μM), and slDNA (2 μM) recorded at 25°C.

To prove motif Ia is necessary and sufficient for the interaction, two additional deletion proteins-MAD28 and MAD8-were purified (Table 2). Binding studies showed that the interaction of ADAADiN with MAD28 does not reach saturation while the inhibitor was able to bind to MAD8 with the same binding affinity as ADAAD, thus we conclude that motif Ia is both necessary and sufficient for the interaction of the inhibitor with the protein (Figure 2A and B).

### Conformation of ADAAD in presence of ADAADi

Theoretically, the ATPase activity of ADAAD could be blocked either by competing for DNA binding or by inducing an ATPase inactive conformation since conformational changes are critical for ATP hydrolysis [13].

EMSA demonstrated that ADAAD-DNA complex could be competed out in the presence of excess cold DNA but not in the presence of excess inhibitor (Figure 2C and D) therein confirming that ADAADi is not a classical competitive inhibitor with respect to DNA.

Further, the accessibility of the buried as well as surface exposed tryptophans to acrylamide, a neutral quencher, was altered when ADAADi binds to ADAAD (Figure 2E). ADAAD alone shows biphasic accessibility (K_SV1_ and K_SV2_) to acrylamide, which reduces significantly in the presence of ADAADi suggesting that both sets of tryptophan residues get further buried in the presence of the inhibitor (Table 3; Figure 2E). Addition of the inhibitor to a protein solution saturated either with ATP or with slDNA resulted in a further drop in both K_SV1_ and K_SV2_ (Table 3). Finally, both K_SV1_ and KSV_2_ decreased further when the protein was saturated with inhibitor, ATP, and slDNA (Table 3; Figure 2E). Comparison of the K_SV_ values of ADAAD-ADAADiN-ATP-slDNA with that of ADAAD-ATP-slDNA indicates an alteration in the conformation of the protein, which was confirmed using CD spectroscopy. In theory, the order of addition of components could result in four possible types of complexes-[E.ATP.I.DNA], [E.I.ATP.DNA], [E.DNA.I.ATP], and [E.I.DNA.ATP] -formed by the interaction of ADAAD (E), ATP, DNA and ADAADi (I) (Figure S4). As shown in Figure 2F, the [E.DNA.ATP] complex is significantly distinct from that of each of the above four complexes.

**Table 3:** Calculation of K_SV1_and K_SV2_ for Protein-ATP-DNA-inhibitor interaction.

	K_SV1_	P value	K_SV2_	P value	f_a_
*Protein	9.76±0.14	<0.0001	6.70±1.0	0.0009	0.95±0.002
1Protein + inhibitor	4.91±0.17	<0.0001	2.89±0.15	<0.0001	0.82±0.034
*Protein + ATP	7.28±0.40	<0.0001	4.25±0.61	0.00045	0.88±0.005
1Protein + ATP + inhibitor	5.4±0.23	<0.0001	3.49±0.33	0.008	0.84±0.021
Protein + inhibitor + ATP	5.22±0.18	<0.0001	3.32±0.31	0.00176	0.85±0.02
*Protein + slDNA	5.73±0.13	<0.0001	3.09±0.17	0.00065	0.85±0.005
1Protein + slDNA + inhibitor	5.63±0.43	0.001	3.78±0.21	0.0004	0.81±0.065
1Protein + inhibitor + slDNA	5.65±0.23	<0.0001	3.45±0.25	0.00083	0.81±0.025
*Protein + slDNA + ATP	5.63±0.5	<0.0001	2.75±0.08	0.00235	0.9±0.06
1Protein + inhibitor + slDNA + ATP	3.2±0.058	<0.0001	2.1±0.12	0.0004	0.78±0.02
Protein + slDNA+ inhibitor + ATP	3.78±0.11	<0.0001	2.39±0.09	0.0014	0.87±0.02
Protein + inhibitor + ATP + slDNA	3.43±0.14	<0.0001	2.17±0.4	0.032	0.82±0.01
Protein + ATP + inhibitor + slDNA	3.1±0.13	<0.0001	1.47±0.09	0.00064	0.73±0.02

* Data reported in Nongkhlaw *et al*. (Nongkhlaw, 2009).

1 Average of at least two independent experiments with each experiment done in duplicates.

With this foundation of biochemical and biophysical observations for the ADAAD/ADAADi interaction, we delineated the effect of ADAADi on neomycin-resistant mammalian cells.

### Neomycin-resistance gene generates ADAADi

**Figure 3:**
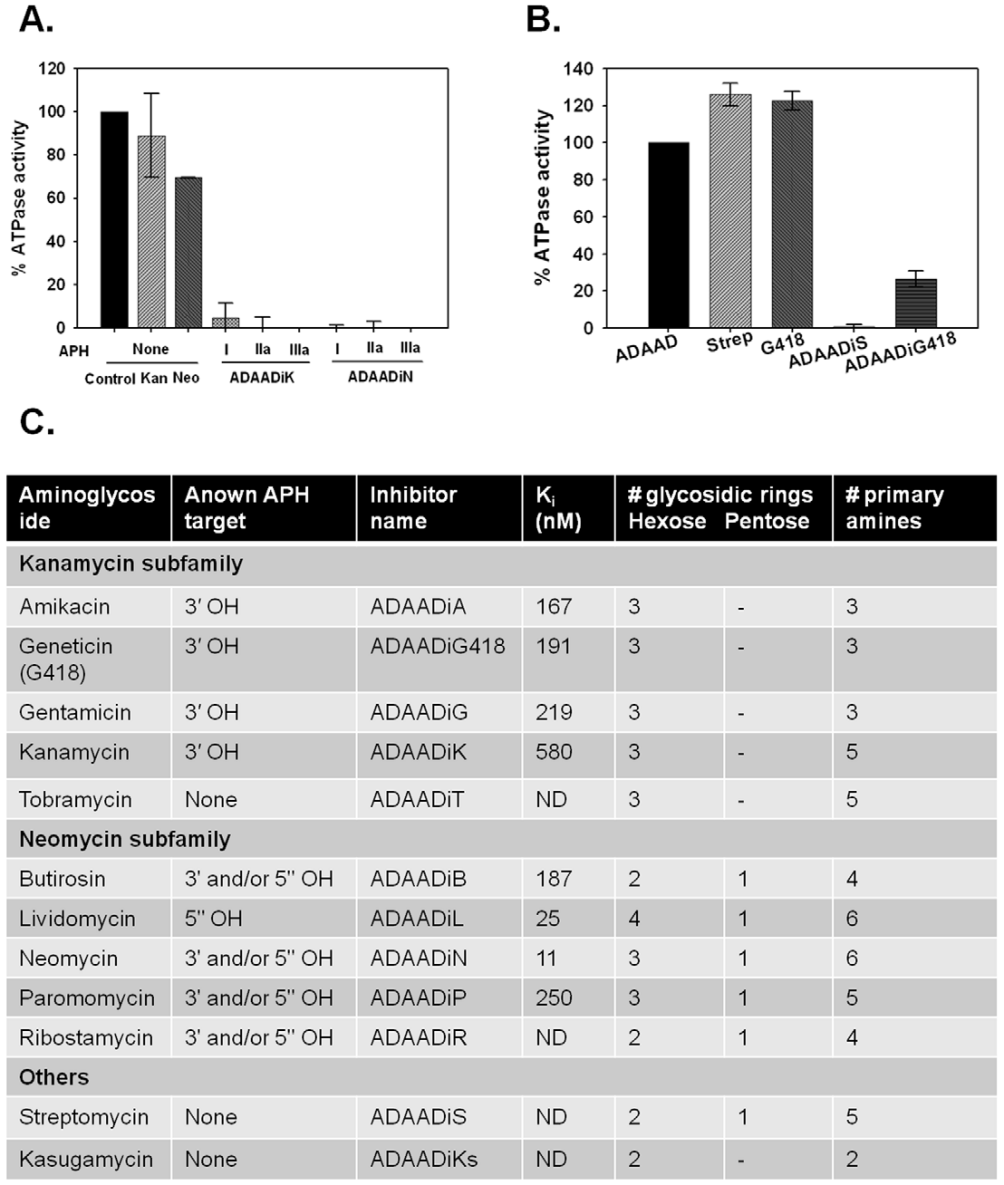
ADAADi formation is catalyzed by different isoforms of APH using different aminoglycoside substrates. (A). APH (3′)-I, APH (3′)-IIa, and APH (3′)-IIIa catalyze ADAADi formation. ADAADi, synthesized by the three isozymes of APH, was purified and ATPase assays with 0.22 μM His-ADAAD and 68 μM kanamycin (Kan), 44 μM neomycin (Neo), 1.6 μM ADAADiK from APH(3′)-I (I) and APH(3′)-IIa (IIa), 1.2 μM ADAADiK from APH(3′)-IIIa (IIIa), 1.6 μM ADAADiN from APH (3′)-I (I), 2 μM ADAADiN from APH(3′)-IIa (IIa) , and 1.4 μM ADAADiN from APH(3′)-IIIa (IIIa) were done as described. (B). **ADAADi is produced from G418 as well as streptomycin** by APH (3′)-IIIa. ATPase assays were done either in the absence or presence of 200 μM streptomycin, 2 μM G418, 4 μM ADAADiS, 4 μM ADAADiG418. (C). ADAADi produced using APH (3′)-IIIa from commercially available aminoglycosides.

Seven different isoforms of APH [7] are known of which APH (3′)-I is used as selection marker for prokaryotic systems, while APH (3′)-IIa (neomycin-resistance gene) is used as the selection marker for eukaryotic systems. Figure 3A shows that APH (3′)-I, APH (3′)-IIa and APH (3′)-IIIa can each catalyze synthesis of ADAADiK and ADAADiN from kanamycin and neomycin, respectively.

In general, aminoglycosides are subdivided into three subfamilies (Figure S5) with a few outliers. Of principle interest are streptomycin, which is used as penicillin-streptomycin (pen-strep) solution to prevent contamination of mammalian cell cultures and G418, which is used as selection reagent for transfected mammalian cells in culture. APH (3′)-IIIa can catalyze ADAADi formation from many aminoglycosides, including G418 and streptomycin (Figure 3B and C). Notably, tobramycin, normally considered an inhibitor of the APH enzymes because it lacks the 3′-hydroxyl for phosphorylation and thus has no previously known product, also yields an ADAADi [16]. Mock syntheses of inhibitor omitting either APH, ATP or aminoglycoside did not yield an inhibitor and therefore the derivation of ADAADi from APH modification of tobramycin suggests a novel synthetic mechanism for ADAADi production.

### *aph (3*′*)-IIa* transfected mammalian cells are resistant to exogenous and endogenous ADAADi

**Figure 4:**
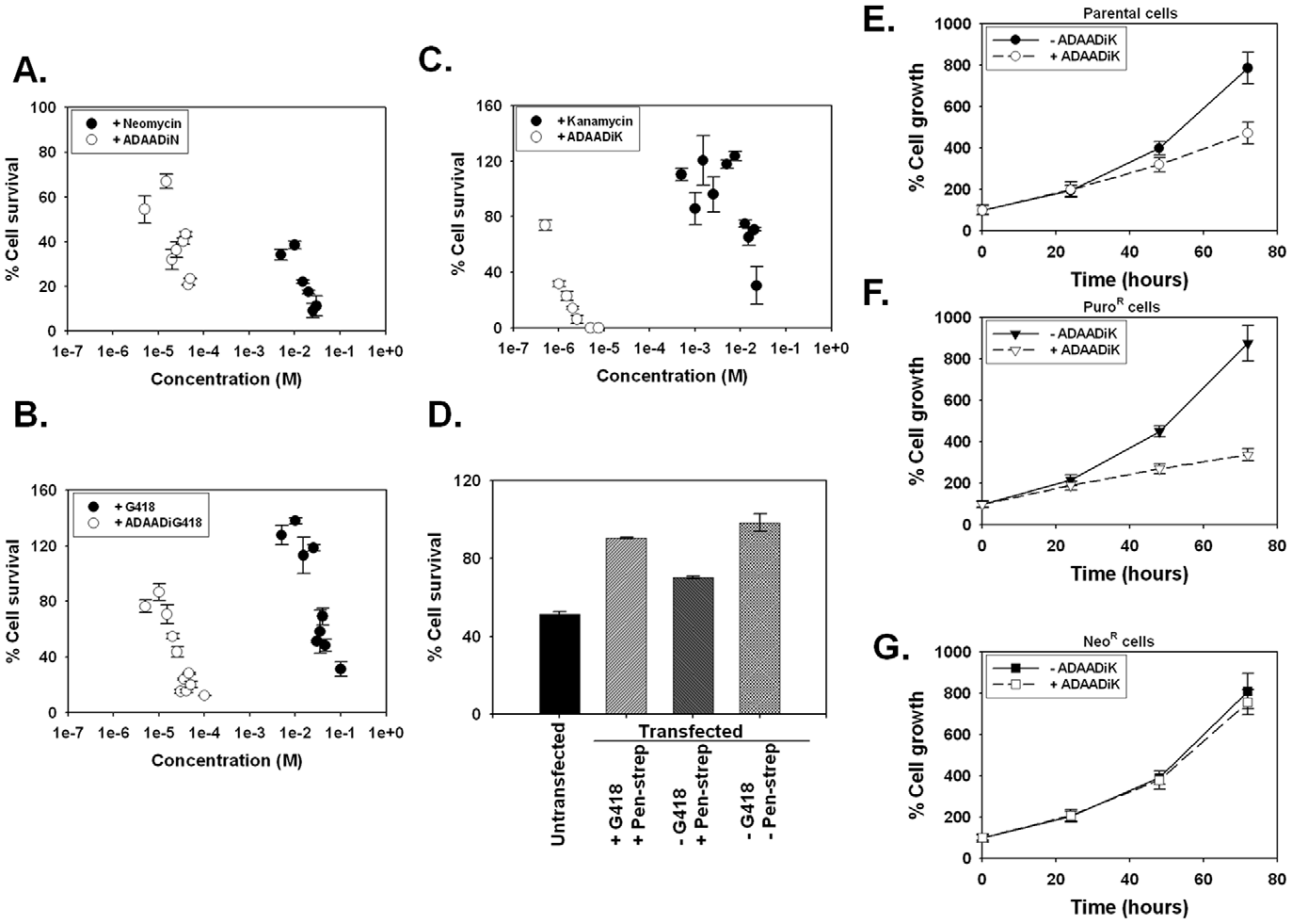
Effect of ADAADi on untransfected and transfected mammalian cells. (A). Untransfected Neuro2A cells treated with neomycin (•) or ADAADiN (○). (B). Untransfected Neuro2A cells treated with G418 (•) or ADAADiG418 (○). (C). Untransfected Neuro2A cells treated with kanamycin (•) or ADAADiK (○). (D). Comparing the effect of 50 μM ADAADiN on untransfected and stably transfected Neuro2A cells grown as indicated post-selection. (E). Parental C2C12 cells grown in the absence (•) and presence (○) of 200 μM ADAADiK. (F). Puromycin-resistant C2C12 cells carrying the pCPLX retroviral vector and grown in the absence (▾) and presence (▿) of 200 μM ADAADiK. (G). G418-resistant C2C12 cells carrying the pLNCX retroviral vector and grown in the absence (▪) and presence (□) of 200 μM ADAADiK.

Mammalian cells (Neuro2A) are sensitive to exogenous application of ADAADi (ADAADiN, ADAADiG418, and ADAADiK) (Figure 4A, B, and C), which is consistent with the fact that some of the SWI2/SNF2 proteins are known to be essential for viability [17].

To study the effect of ADAADi on neomycin-resistant cell lines, we stably transfected Neuro2A cells with pcDNA 3.1 myc/his (-) vector harbouring *aph (3*′*)-IIa* (Figure S6). After transfection with vector the cells were selected in presence of 400 μg/ml of G418 and pen-strep until stable transfectants were obtained. The stable transfectants were maintained in presence of 100 μg/ml of G418 and pen-strep. To study the effect of ADAADi, cells were grown under three conditions. In one condition cells were grown in the presence of pen-strep as well as 400 μg/mlG418 (labeled as antibiotics) for 24 hours. This condition allowed us to understand what happens when cells were constantly selected in the presence of antibiotics and therefore, generated ADAADi. In the second condition, we grew the cells in the presence of pen-strep but in the absence of G418 for 24 hours. This condition enabled us to understand whether streptomycin by itself can generate sufficient amount of ADAADi. In the third condition, we grew the cells in the absence of both G418 and pen-strep for 24 hours. This enabled us to understand what happens when ADAADi is not produced inside the cell. This protocol was adhered in all the studies described hereafter.

Surprisingly, we found that the Neuro2A cell line became resistant to exogenous 50 μM ADAADiN following selection of cells stably transfected with the pcDNA 3.1 myc/his (-) vector harbouring *aph (3*′*)-IIa* and grown in the presence of antibiotics (Figure 4D). Further, the transfected cells continued to be resistant to exogenous ADAADiN even after removal of the antibiotics post-selection (Figure 4D).

These findings were corroborated using a myoblast cell line (C2C12) along with two matching cell lines derived by introducing either neomycin-resistance or puromycin-resistance to the parental cell line via the retroviral vector (pLNCX/pLPCX). The vectors are identical with the exception of the resistance cassette and neither of the derived cell lines (or vectors) carried any additional DNA sequences for expression and thus should be genetically identical with the exception of the differing mechanisms of antibiotic resistance. The C2C12 parental (Figure 4E) and C2C12 puromycin-resistant (Figure 4F) cell lines were sensitive to ADAADiK but the C2C12 neomycin-resistant cell line was ADAADiK-resistant (Figure 4G).

### SWI2/SNF2 proteins are inactivated in *aph (3*′*)-IIa* transfected mammalian cells

**Figure 5:**
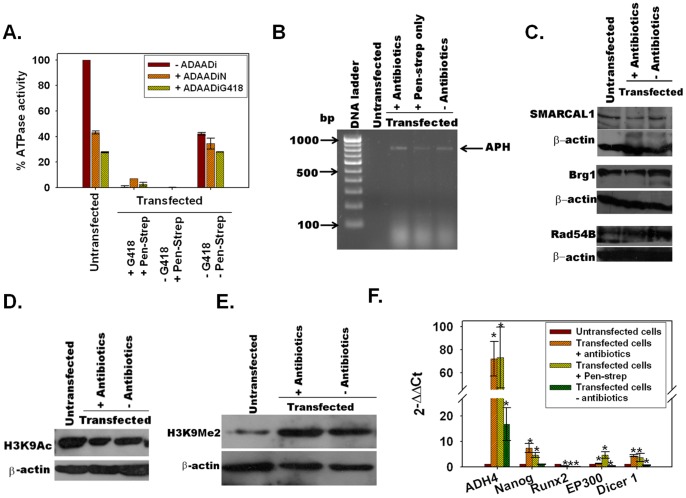
Production of ADAADi in Neuro2A cells leads to alterations in the epigenome. (A). Comparing the *in vivo* ATPase activity of SMARCAL1 present in untransfected Neuro2A cells and stably transfected Neuro2A cells grown as indicated post-selection. (B). APH transcript expression in untransfected and transfected Neuro2A cells. (C). SWI2/SNF2 expression in untransfected and stably transfected Neuro2A cells grown as indicated was analysed using polyclonal anti-SMARCAL1 antibody, anti-Brg1 antibody, and anti- Rad54B antibody. (D). Western blot analysis of H3K9Ac levels in untransfected and stably transfected Neuro2A cells. (E). Western blot analysis of H3K9Me2 levels in untransfected and stably transfected Neuro2A cells grown as indicated by western blot. β-actin was used as loading control in these experiments. (F). The levels of ADH4, Nanog, Runx2, EP300, and Dicer1 was estimated by quantitative RT-PCR. The transcript levels in stably transfected cells were calculated with respect to the levels present in untransfected cells. The data are an average of two independent experiments, each experiment done in duplicate. Error bars indicate standard deviation and stars indicate statistical significance at P<0.05. The P-values are given in Table S3.

Resistance to ADAADi connotes inactivation of SWI2/SNF2 proteins. As shown in Figure 5A, immunoprecipitated SMARCAL1, the mouse orthologue of ADAAD [18], from untransfected Neuro2A cells was active and responsive to ADAADiN and ADAADiG418, while the protein immunoprecipitated from transfected Neuro2A cells cultured post-selection either in the presence of both antibiotics or in the presence of pen-strep alone was inactive. The activity of SMARCAL1 was partially restored when the cells were grown in the absence of antibiotics for 24 hours. This was not due to loss of APH transcript (Figure 5B) and therefore was attributed to cessation of ADAADi synthesis.

Western blot analysis showed that there was a marginal reduction in the levels of SMARCAL1 (Figure 5C). In addition, the levels of Brg1, a chromatin remodeler required for active transcription [19] was also found to be slightly downregulated. However, the levels of Rad54B, a SWI2/SNF2 protein required for double-strand break repair [20], [21], was found to be unaltered (Figure 5C). The subcellular localization of Brg1 as well as SMARCAL1, however, was found unaltered in the absence and presence of antibiotics (Figure S7A and B).

### Alterations in epigenome and gene expression levels

Reduced levels of functional SWI2/SNF2 proteins such as ADAAD (SMARCAL1) lead to the hypothesis that there must be compensatory epigenetic changes occurring in order for stably transfected cells to survive the resultant production of ADAADi in the presence of aminoglycoside antibiotics. Consequently, we investigated the levels of H3K9 acetylation (H3K9Ac), associated with transcription initiation [22], and H3K9 dimethylation (H3K9Me2), associated with transcription repression [22], in *aph* transfected cells.

H3K9Ac was found to be downregulated in *aph (3*′*)-IIa* transfected cells when compared to the untransfected cells (Figure 5D). Concomitantly, the transfected cells showed an increase in the level of H3K9Me2 as compared to the untransfected cells (Figure 5E), confirming alterations in histone modifications.

As a corollary of the altered epigenetics, a microarray analysis using an Agilent platform showed that the transcription of many tissue-specific genes as well as metabolic enzymes was altered in vector transfected cell lines (Figure S8). Specifically, 2706 genes were upregulated and an equal number were downregulated (GEO accession number GSE36142). The microarray data corroborated a previous report that transfection of neomycin-resistance gene into NIH3T3 cells results in decrease in expression of procollagen1α as well as fibronectin genes [23].

We further validated the microarray data for five genes, Nanog, ADH4, Runx2, Dicer1, and EP300, using quantitative RT-PCR. The microarray data showed Nanog and ADH4 were upregulated while Runx2, Dicer1, and EP300 were downregulated. These findings are consistent with the reported siRNA-mediated downregulation of Brg1 yields upregulated Nanog levels [24].

In stably transfected cells grown either in the presence of both the selective aminoglycoside (G418) and streptomycin or in the absence of G418 but presence of pen-strep alone, Nanog and ADH4 transcript levels were upregulated while Runx2 transcript levels were downregulated validating the microarray results. However, the regulation of Dicer1and EP300 levels could not be verified (Figure 5F). These data confirms that ADAADi produced from the streptomycin in pen-strep is sufficient to maintain the epigenetic alterations.

Further, the transcript levels of Nanog were restored to the untransfected levels while ADH4 were partially restored to untransfected levels when transfected cells were grown in the absence of antibiotics for 24 hours. However, the transcript levels of EP300, Runx2, and Dicer1 were further downregulated in these cells (Figure 5F).

Thus, epigenetic alterations and changes in gene expression pattern are prominent features of these *aph* transfected cells which are commonly labeled as neomycin-resistant.

### Brg1 recruitment to *sg2na* promoters is impaired

SG2NA, a member of the Striatin sub-family containing WD-40 repeats, plays a role cell signaling as well as vesicular trafficking [25], [26] and is known to exist as multiple splice variants [27]. Microarray analysis corroborated our observation that SG2NA is repressed in *aph* transfected Neuro2A cells. Therefore, SG2NA was used as the test gene to understand the transcription repression mediated by ADAADi in stably transfected cells.

**Figure 6:**
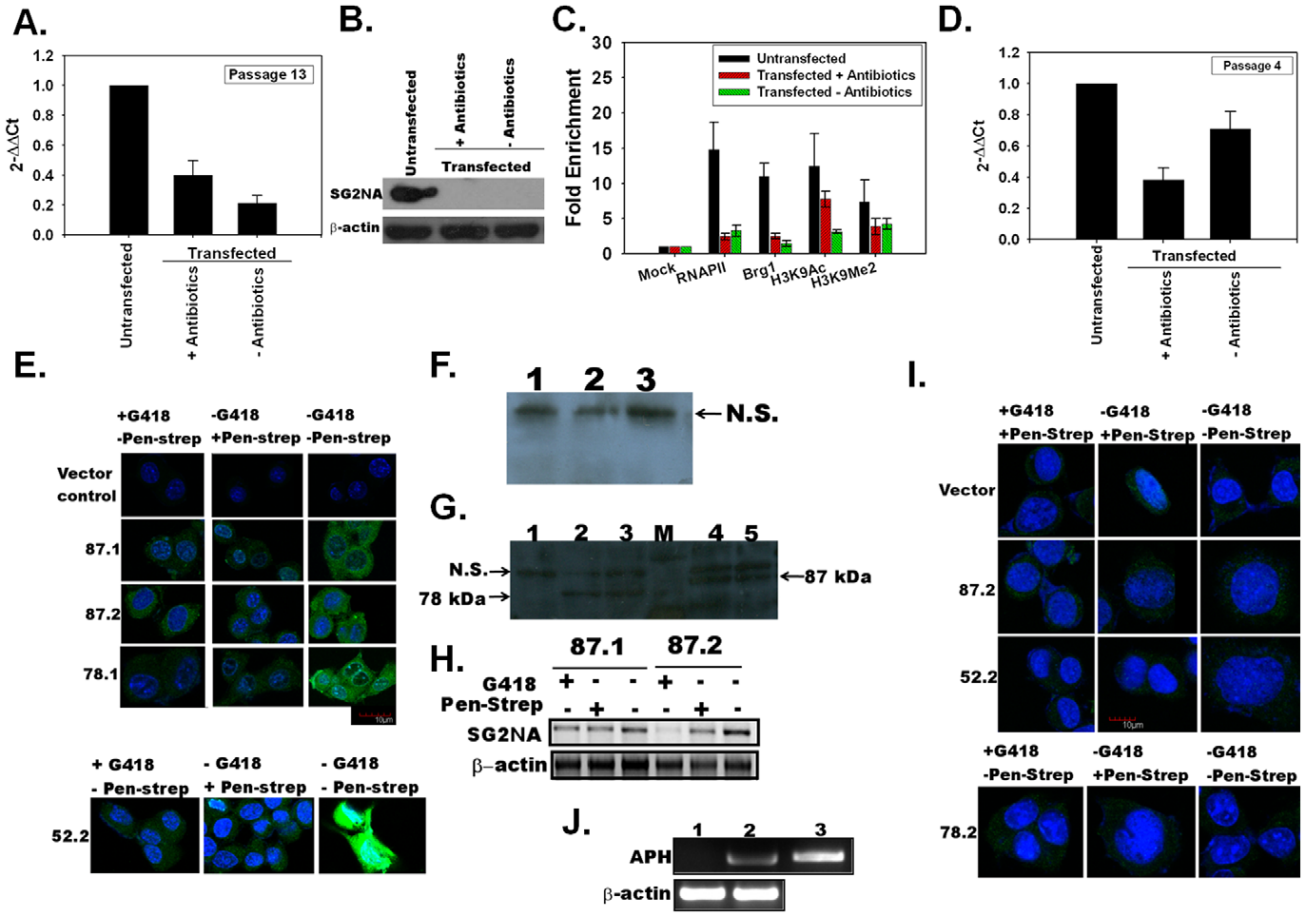
Expression of endogenous SG2NA is influenced by ADAADi production. The transcript as well as protein expression was monitored in the untransfected cells as well as in cells stably transfected with pcDNA 3.1 myc/his (−) vector at passage 13. (A). Endogenous SG2NA transcript was analyzed by quantitative RT-PCR in untransfected and transfected Neuro 2Acells. (B). Endogenous SG2NA protein analyzed by western blot using antibody against SG2NA. (C). *sg2na* promoter occupancy by RNAPII, Brg1, H3K9Ac, and H3K9Me2 was analysed in untransfected and transfected Neuro 2A cells using ChIP. Fold enrichment was calculated with respect to the mock ChIP done using IgG antibodies. (D). SG2NA transcript level in transfected cells at passage 4. (E). **Expression of exogenous SG2NA expressed using pcDNA 3.1 myc/his (−) vector** **is also influenced by ADAADi production.** Overexpression of three variants of SG2NA in Neuro2A cells were monitored using anti-myc antibody. Transfected cells (passage 4) were grown as indicated for 12 hours before analysis. Two clones of 87 kDa (87.1 and 87.2), one clone each of 78 kDa (78.1) and of 52 kDa (52.2) were analyzed for protein expression. The cells transfected with vector alone were used as control. Protein expression was observed only when cells were grown in the absence of both antibiotics. (F). Western blot analysis of expression of 87- and 78-kDa proteins in clones 87.1 and 78.1 in stably transfected cells grown in the presence of antibiotics using anti-myc antibody. Lane 1: vector alone transfected cells; Lane 2: 78.1 clone; Lane 3: 87.1 clone. N.S., indicating non-specific band, was used as loading control. (G). The expression of 78- and 87- kDa protein in 78.1, 78.2, 87.1 and 87.2 clones was monitored in stably transfected cells grown in the absence of antibiotics. Lane 1: vector transfected cells; Lane 2: 78.1 clone; Lane 3: 78.2 clone; M: marker; Lane 4: 87.1 clone; Lane 5: 87.2 clone. N.S., indicating non-specific band, was used as loading control. (H). Semi-quantitative RT-PCR analysis done using insert-specific forward primer and vector-specific reverse primer confirms that 87 kDa transcript expression is observed only when the cells are grown the absence of antibiotics. (I). The expression of 87 kDa, 78 kDa, and 52 kDa was not observed in stably transfected cells even after removal of antibiotics when the cells were freeze-thawed at passage 9. (J). Semi-quantitative RT-PCR analysis of APH transcript. Lane1: Untransfected Neuro2A cells; Lane 2: Stably transfected Neuro2A cells; Lane 3: control reaction using purified pcDNA 3.1 myc/his (−) vector.

As shown in Figure 6A, the transcript levels of endogenous SG2NA were downregulated in Neuro2A cells *aph* transfected cells, grown either in the absence or in the presence of antibiotics. Western blot analysis corroborated this observation (Figure 6B).

Next, ChIP analysis showed that the RNAP II as well as Brg1 were present in the promoter region of *sg2na* in untransfected control cells but not in the stably transfected cells grown either in the presence or in the absence of both antibiotics (Figure 6C). However, the levels of H3K9Me2 and H3K9Ac were not found to be statistically different between transfected cells and untransfected cells (Figure 6C).

Thus, at the *sg2na* promoter, Brg1 levels correlate with the recruitment of RNAPII in untransfected cells. In stably transfected cells, lower Brg1 levels correlate with reduced RNAPII yielding apparent transcriptional repression.

### Epigenome alteration is quantized

The above experimental data suggest that the epigenome is altered in *aph* transfected cells. We next sought to ask whether this alteration occurs soon after selection or whether it required subsequent cell passages.

At passage 4, after selection, the endogenous SG2NA expression (in absence of antibiotics) was 80% of that for the untransfected whereas at passage 13 it was only 20% (compare Figure 6A and D) leading to the conclusion that gene expression can be modulated in earlier passages.

A similar observation was recorded when three variants (87, 78, and 52) of SG2NA were overexpressed as Myc-fusion proteins using pcDNA 3.1 myc/his (−) vector in Neuro2A cells. Immunocytochemistry experiments showed that stably transfected cells grown in the presence of G418 or streptomycin did not express these variants; however, when the antibiotics were removed for 12 hours protein expression was observed (Figure 6E). This was further confirmed by western blot (Figure 6F and G). Semi-quantitative RT-PCR for 87 kDa transcript in 87.1 and 87.2 clones also corroborated the observation (Figure 6H). Thus, not only is the overexpression of genes affected in *aph* transfected cells but also the effect of ADAADi is reversible in the initial passages.

However, when the cells were frozen at passage 9 and subsequently thawed it was found that the effect of ADAADi was no longer reversible. The immunofluorescence assay showed that the expression of overexpressed SG2NA variants was not responsive to the removal of antibiotics from the growth media for 12 hours (Figure 6I). Semi-quantitative RT-PCR confirmed that the *aph (3*′*)-IIa* was transcribed in these cells (Figure 6J) and therefore cellular changes could not be attributed to loss of the transfected vector. These data confirm that after several passages the epigenetic changes that have occurred within the cell cannot be reversed by removing the antibiotics for 12–24 hours.

## Discussion

Southern and Berg in 1982 showed that prokaryotic APH genes could be used for transfecting eukaryotic cells [10] and the methodology has subsequently been widely adopted both for *in vitro* and *in vivo* studies. However, there are two widely acknowledged problems: i) variable expression from the same vector, vector instability and low titres [28], [29]; and ii) *neo* resistance gene induces changes within the cell [23].

The APH enzyme inactivates aminoglycosides and in the process generates a molecule, ADAADi, which is a potent inhibitor of the eukaryotic SWI2/SNF2 proteins. ADAADi is unique as it is neither an ATP competitor nor DNA competitor; instead it binds to a region within motif Ia inducing an ATPase incompetent conformation in the ATP-dependent remodeling protein. Given the wide variability in motif Ia of the SWI2/SNF2 proteins, ADAADi is an exciting discovery for it provides hope for generating species (orthologue)-specific as well as protein (homolog)-specific inhibitors for this class of chromatin remodelers.

Further, ADAADi provides an unequivocal explanation for the problems associated with mammalian cell transfections when employing neomycin-resistance gene-based vectors. Transfecting mammalian cells with these vectors and subsequent selection with G418/neomycin catalyzes ADAADi formation, which inactivates cellular SWI2/SNF2 proteins. To survive under these circumstances, a cell modifies its epigenome and thus, its transcriptome. In other words, the selection process ensures selection of only those cells that have acquired epigenetic changes to survive. Our results lead us to postulate that every *aph* transfected cell that survives has an altered epigenome and transcriptome.

The redefinition of the epigenome appears to be quantized. Thus, in initial stages, just after selection of stable cell lines in the presence of G418, the epigenetic alterations can be reversed by removing aminoglycosides from the growth media for 12 hours. However, as the cells continue to be grown in the presence of antibiotics, removing the antibiotics from the growth media even for 24 hours can no longer reverse the alterations occurring within a cell even though the activity of the SWI2/SNF2 proteins are partially restored. That is, the epigenetic changes persist after significant periods of selective pressure and this is reminiscent of recently reported epigenetic alterations in cancer cells, where subpopulations of cancer cells with altered drug tolerance were shown to spontaneously emerge due to altered histone methylation [30]. The drug tolerance state was reversible; however, it took 8 passages to reverse the status of the cell. Although we have not explored reversal, we acknowledge that epigenetic alterations in ADAADi resistant cells might possibly be reversed after sufficient passaging either in the absence of antibiotics or after removal of the resistant cassette.

Our observations raise a plethora of questions and hypotheses. For instance, do all cells transformed with plasmid containing a neomycin-resistance gene possess the same kind of epigenetic alterations or is there variability in the alterations? Given the fact that the epigenome differs between cell types, there is no *a priori* reason to believe that every cell type will have the same epigenetic alteration. It is also possible that epigenetic variations exists between clones derived from same cell giving raise to clonal heterogeneity, which would account for the notorious difficulties commonly observed with onco-retroviral vectors in gene therapy [28].

The use of “neo cassettes” and APH has been an unquestionable asset in redefining eukaryotic molecular biology. However, it is time for careful reflection and analysis of data as we recognize that the data generated using the plasmid containing APH could include heretofore unexplained and often unforeseen changes to the systems regulating chromatin structure.

## Supporting Information

Figure S1 **Binding of ADAADi to ADAAD in absence of ATP and slDNA.** (A). The binding constant for the interaction of ADAADiN with ADAAD was calculated using fluorescence spectroscopy. (B). The binding constant for the interaction of ADAADiK with ADAAD was calculated using fluorescence spectroscopy. In the absence of both ATP and slDNA, assuming a single binding site for the inhibitor, the K_d_ for the interaction of ADAADiN with ADAAD was estimated to be 35.8±5.0 nM, while the K_d_ for ADAADiK interaction with ADAAD was calculated to be 29.2±4.2 nM.
(TIFF)

Figure S2 **Binding of ADAADiN, ATP, and slDNA to ADAAD.** (A). Binding of ADAADiN to ADAAD in the presence of 40 μM ATP. (B). Binding of ADAADiN to ADAAD in presence of 3 μM slDNA. (C). Binding of ATP in the presence of 2 μM ADAADiN. The K_d_ for the interaction was calculated to be 0.1±0.003 μM, suggesting that in the presence of ADAADiN, ATP binds to the protein with 10-fold higher affinity than in the absence of the inhibitor. (D). Binding of slDNA to ADAAD in the presence of 2 μM inhibitor. The K_d_ was calculated to be 0.63±0.1 nM, again suggesting that slDNA binds with higher affinity to the protein in the presence of ADAADiN.
(TIFF)

Figure S3 **Binding of ADAADiK, ATP, and slDNA to ADAAD.** (A). Binding of ADAADiK to ADAAD in the presence of 40 μM ATP. (B) Binding of ADAADiK to ADAAD in presence of 3 μM slDNA. (C). Binding of ATP in the presence of 2 μM ADAADiK. The K_d_ for the interaction was calculated to be 0.1±0.01 μM, suggesting that in the presence of ADAADiK, ATP binds to the protein with 10-fold higher affinity than in the absence of the inhibitor. (D). Binding of slDNA to ADAAD in the presence of 2 μM inhibitor. The K_d_ was calculated to be 0.46±0.11 nM, again suggesting that slDNA binds with higher affinity to the protein in the presence of ADAADiN.
(TIFF)

Figure S4 **Model for the interaction of ATP, stem-loop DNA, and ADAADiN with ADAAD**. ADAAD (E) can interact with ADAADi (I) in the absence of both ATP and DNA to form a binary complex [EI]. This complex can further interact either with ATP or DNA, such that these ligands bind to the protein with higher affinity. The ternary complex, [E.I.ATP] or [E.I.DNA], can subsequently interact with DNA or ATP but this interaction does not lead to ATP hydrolysis, presumably because the conformation of the complex does not allow ATP to be hydrolyzed.
(TIFF)

Figure S5 **Structure of aminoglycosides**. The kanamycin sub family consists of kanamycin, tobramycin, and G418. The neomycin sub-family consists of neomycin, lividomycin, and ribostamycin. Others like streptomycin and kasugamycin lack the central deoxystreptidine ring.
(TIFF)

Figure S6 **Creating stable aph transfected cell lines and assay conditions.** Mouse Neuro2A cells were transfected with pcDNA 3.1 myc/his (−) using Lipofectamine (Life Technologies). After transfection cells were selected in the presence of 400 μg/ml G418 in the growth media till clones were obtained. Single clones were transferred to new plate and maintained in the presence of 100 μg/ml G418 in the growth media. For studying the effect of ADAADi produced inside the cells by the action of vector-encoded APH enzyme, cells were grown for 24 hours, prior to assay, as follows: i) in the presence of 400 μg/ml G418 and pen-strep; ii) in the absence of G418 but presence of pen-strep; iii) in the absence of both G418 and pen-strep. The same protocol was used for analyzing the expression of SG2NA variants. The only exception was that the cells were grown for 12 hours, prior to assay, as follows: i) in the presence of 400 μg/ml G418 and pen-strep; ii) in the absence of G418 but presence of pen-strep; iii) in the absence of both G418 and pen-strep.
(TIFF)

Figure S7 **Localization of SWI2/SNF2 proteins is not altered in transfected cells.** (**A**)**.** Localization of SMARCAL1 in untransfected (top) and stably transfected (bottom pairs) Neuro2A cells. Following selection of a stable transfectant, the cells were grown either in the presence or absence of antibiotics and studied using polyclonal antibiodies raised against the N-terminal region of SMARCAL1. (B). Localization of Brg1 in untransfected and stably transfected Neuro2A cells grown either in the presence or absence of antibiotics was studied using monoclonal antibody against Brg1. The secondary antibody in both cases was conjugated to TRITC and the nucleus was stained using Hoechst.
(TIFF)

Figure S8 **Gene expression is altered in stably transfected Neuro2A cells.** The gene expression in transfected Neuro2A cells grown in the presence of antibiotics was compared with the the expression profile in untransfected Neuro2A cells. The number indicates the number of genes upregulated or downregulated.
(TIFF)

Table S1 List of primers used for RT-PCR and ChIP analysis.
(DOC)

Table S2 Primers used for amplifying *aph (3*′*)-I* and *aph (3*′*)-IIa* genes. *aph (3*′*)-I* was amplified from pET 28a (+) and *aph (3*′*)-IIa* was amplified from pCDNA 3.1 vector.
(DOC)

Table S3 P-values calculated for RT-PCR data showing alteration in expression of ADH4, Nanog, RUNX2, EP400 and Dicer1.
(DOC)

## References

[1] Hargreaves DC, Crabtree GR (2011) ATP-dependent chromatin remodeling: genetics, genomics and mechanisms. Cell Res 21: 396–420 doi:10.1038/cr.2011.32. 21358755 10.1038/cr.2011.32 PMC3110148

[2] Morettini S, Podhraski V, Lusser A (2008) ATP-dependent chromatin remodeling enzymes and their various roles in cell cycle control. Front Biosci 13: 5522–5532. 18508602 10.2741/3096

[3] Clapier CR, Cairns BR (2009) The biology of chromatin remodeling complexes. Annu Rev Biochem 78: 273–304 doi:10.1146/annurev.biochem.77.062706.153223. 19355820 10.1146/annurev.biochem.77.062706.153223

[4] Fourmy D, Recht MI, Blanchard SC, Puglisi JD (1996) Structure of the A Site of Escherichia coli 16S Ribosomal RNA Complexed with an Aminoglycoside Antibiotic. Science 274: 1367–1371 doi:10.1126/science.274.5291.1367. 8910275 10.1126/science.274.5291.1367

[5] Vicens Q, Westhof E (2001) Crystal Structure of Paromomycin Docked into the Eubacterial Ribosomal Decoding A Site. Structure 9: 647–658 doi:10.1016/S0969-2126(01)00629–3. 11587639 10.1016/s0969-2126(01)00629-3

[6] Kotra LP, Haddad J, Mobashery S (2000) Aminoglycosides: Perspectives on Mechanisms of Action and Resistance and Strategies to Counter Resistance. Antimicrobial Agents and Chemotherapy 44: 3249–3256 doi:10.1128/AAC.44.12.3249–3256.2000. 11083623 10.1128/aac.44.12.3249-3256.2000 PMC90188

[7] Shaw KJ, Rather PN, Hare RS, Miller GH (1993) Molecular genetics of aminoglycoside resistance genes and familial relationships of the aminoglycoside-modifying enzymes. Microbiol Rev 57: 138–163. 8385262 10.1128/mr.57.1.138-163.1993 PMC372903

[8] Wright GD, Thompson PR (1999) Aminoglycoside phosphotransferases: proteins, structure, and mechanism. Front Biosci 4: D9–21. 9872733 10.2741/wright

[9] Wright G (1999) Aminoglycoside-modifying enzymes. Current Opinion in Microbiology 2: 499–503 doi:10.1016/S1369-5274(99)00007–7. 10508725 10.1016/s1369-5274(99)00007-7

[10] Southern PJ, Berg P (1982) Transformation of mammalian cells to antibiotic resistance with a bacterial gene under control of the SV40 early region promoter. J Mol Appl Genet 1: 327–341. 6286831

[11] Muthuswami R, Mesner LD, Wang D, Hill DA, Imbalzano AN, et al (2000) Phosphoaminoglycosides inhibit SWI2/SNF2 family DNA-dependent molecular motor domains. Biochemistry 39: 4358–4365. 10757984 10.1021/bi992503r

[12] Felle M, Exler JH, Merkl R, Dachauer K, Brehm A, et al (2010) DNA sequence encoded repression of rRNA gene transcription in chromatin. Nucleic Acids Research 38: 5304–5314 doi:10.1093/nar/gkq263. 20421213 10.1093/nar/gkq263 PMC2938192

[13] Nongkhlaw M, Dutta P, Hockensmith JW, Komath SS, Muthuswami R (2009) Elucidating the mechanism of DNA-dependent ATP hydrolysis mediated by DNA-dependent ATPase A, a member of the SWI2/SNF2 protein family. Nucleic Acids Res 37: 3332–3341 doi:10.1093/nar/gkp178. 19324887 10.1093/nar/gkp178 PMC2691824

[14] Nongkhlaw M, Gupta M, Komath SS, Muthuswami R (2012) Motifs Q and I are required for ATP hydrolysis but not for ATP binding in SWI2/SNF2 proteins. Biochemistry 51: 3711–3722 doi:10.1021/bi2014757. 22510062 10.1021/bi2014757

[15] Yaffe D, Saxel O (1977) Serial passaging and differentiation of myogenic cells isolated from dystrophic mouse muscle. Nature 270: 725–727. 563524 10.1038/270725a0

[16] McKay GA, Wright GD (1995) Kinetic mechanism of aminoglycoside phosphotransferase type IIIa. Evidence for a Theorell-Chance mechanism. J Biol Chem 270: 24686–24692. 7559583 10.1074/jbc.270.42.24686

[17] Bultman S, Gebuhr T, Yee D, La Mantia C, Nicholson J, et al (2000) A Brg1 Null Mutation in the Mouse Reveals Functional Differences among Mammalian SWI/SNF Complexes. Molecular Cell 6: 1287–1295 doi:10.1016/S1097–2765(00)00127–1. 11163203 10.1016/s1097-2765(00)00127-1

[18] Coleman MA, Eisen JA, Mohrenweiser HW (2000) Cloning and characterization of HARP/SMARCAL1: a prokaryotic HepA-related SNF2 helicase protein from human and mouse. Genomics 65: 274–282 doi:10.1006/geno.2000.6174. 10857751 10.1006/geno.2000.6174

[19] Trotter KW, Archer TK (2008) The BRG1 transcriptional coregulator. Nucl Recept Signal 6: e004 doi:10.1621/nrs.06004. 18301784 10.1621/nrs.06004 PMC2254329

[20] Swagemakers SMA (1998) The Human Rad54 Recombinational DNA Repair Protein Is a Double-stranded DNA-dependent ATPase. Journal of Biological Chemistry 273: 28292–28297 doi:10.1074/jbc.273.43.28292. 9774452 10.1074/jbc.273.43.28292

[21] Kanaar R, Troelstra C, Swagemakers SMA, Essers J, Smit B, et al (1996) Human and mouse homologs of the Saccharomyces cerevisiae RAD54 DNA repair gene: evidence for functional conservation. Current Biology 6: 828–838 doi:10.1016/S0960-9822(02)00606–1. 8805304 10.1016/s0960-9822(02)00606-1

[22] Wang Z, Zang C, Rosenfeld JA, Schones DE, Barski A, et al (2008) Combinatorial patterns of histone acetylations and methylations in the human genome. Nature Genetics 40: 897–903 doi:10.1038/ng.154. 18552846 10.1038/ng.154 PMC2769248

[23] Valera A, Perales JC, Hatzoglou M, Bosch F (1994) Expression of the neomycin-resistance (neo) gene induces alterations in gene expression and metabolism. Hum Gene Ther 5: 449–456 doi:10.1089/hum.1994.5.4–449. 7914094 10.1089/hum.1994.5.4-449

[24] Kidder BL, Palmer S, Knott JG (2009) SWI/SNF-Brg1 Regulates Self-Renewal and Occupies Core Pluripotency-Related Genes in Embryonic Stem Cells. Stem Cells 27: 317–328 doi:10.1634/stemcells.2008–0710. 19056910 10.1634/stemcells.2008-0710

[25] Moreno CS (2000) WD40 Repeat Proteins Striatin and S/G2 Nuclear Autoantigen Are Members of a Novel Family of Calmodulin-binding Proteins That Associate with Protein Phosphatase 2A. Journal of Biological Chemistry 275: 5257–5263 doi:10.1074/jbc.275.8.5257. 10681496 10.1074/jbc.275.8.5257 PMC3505218

[26] Baillat G, Moqrich A, Castets F, Baude A, Bailly Y, et al (2001) Molecular cloning and characterization of phocein, a protein found from the Golgi complex to dendritic spines. Mol Biol Cell 12: 663–673. 11251078 10.1091/mbc.12.3.663 PMC30971

[27] Sanghamitra M, Talukder I, Singarapu N, Sindhu KV, Kateriya S, et al (2008) WD-40 repeat protein SG2NA has multiple splice variants with tissue restricted and growth responsive properties. Gene 420: 48–56 doi:10.1016/j.gene.2008.04.016. 18571342 10.1016/j.gene.2008.04.016

[28] Madigan C, Malik P (2006) Pathophysiology and therapy for haemoglobinopathies; Part I: sickle cell disease. ERM 8. 10.1017/S1462399406010659 16690007

[29] Fiordalisi JJ, Johnson RL, Ulkü AS, Der CJ, Cox AD (2001) Mammalian expression vectors for Ras family proteins: generation and use of expression constructs to analyze Ras family function. Meth Enzymol 332: 3–36. 11305105 10.1016/s0076-6879(01)32189-4

[30] Sharma SV, Lee DY, Li B, Quinlan MP, Takahashi F, et al (2010) A Chromatin-Mediated Reversible Drug-Tolerant State in Cancer Cell Subpopulations. Cell 141: 69–80 doi:10.1016/j.cell.2010.02.027. 20371346 10.1016/j.cell.2010.02.027 PMC2851638

