# Supplementary figures and images for: Global Epigenetic Changes Induced by SWI2/SNF2 Inhibitors Characterize Neomycin-Resistant Mammalian Cells
Source: PLoS One. 2012 Nov 28;7(11):e49822. doi: 10.1371/journal.pone.0049822 (PMC3509132; doi:10.1371/journal.pone.0049822)

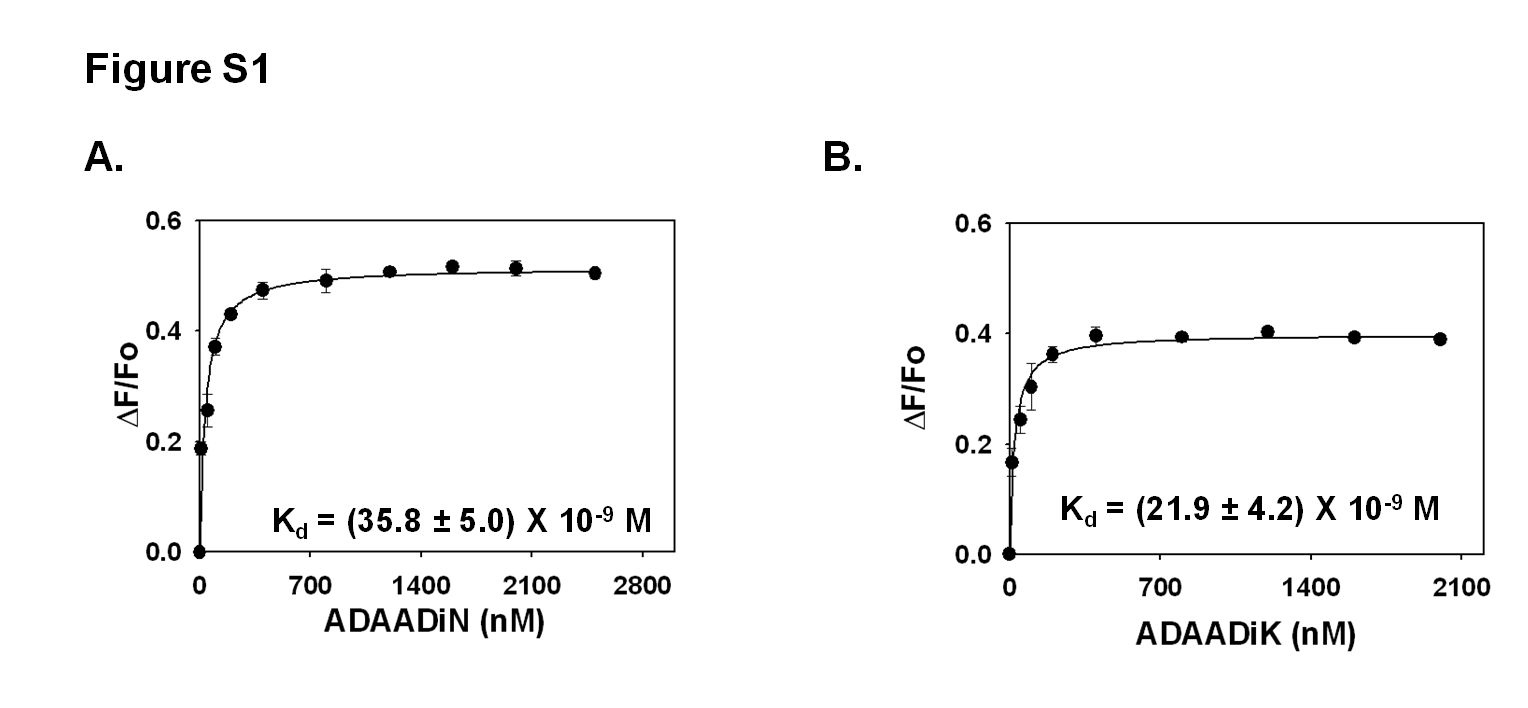

Supplement: Figure S1 — Binding of ADAADi to ADAAD in absence of ATP and slDNA. (A). The binding constant for the interaction of ADAADiN with ADAAD was calculated using fluorescence spectroscopy. (B). The binding constant for the interaction of ADAADiK with ADAAD was calculated using fluorescence spectroscopy. In the absence of both ATP and slDNA, assuming a single binding site for the inhibitor, the Kd for the interaction of ADAADiN with ADAAD was estimated to be 35.8±5.0 nM, while the Kd for ADAADiK interaction with ADAAD was calculated to be 29.2±4.2 nM. (TIFF) [file pone.0049822.s001.tiff]

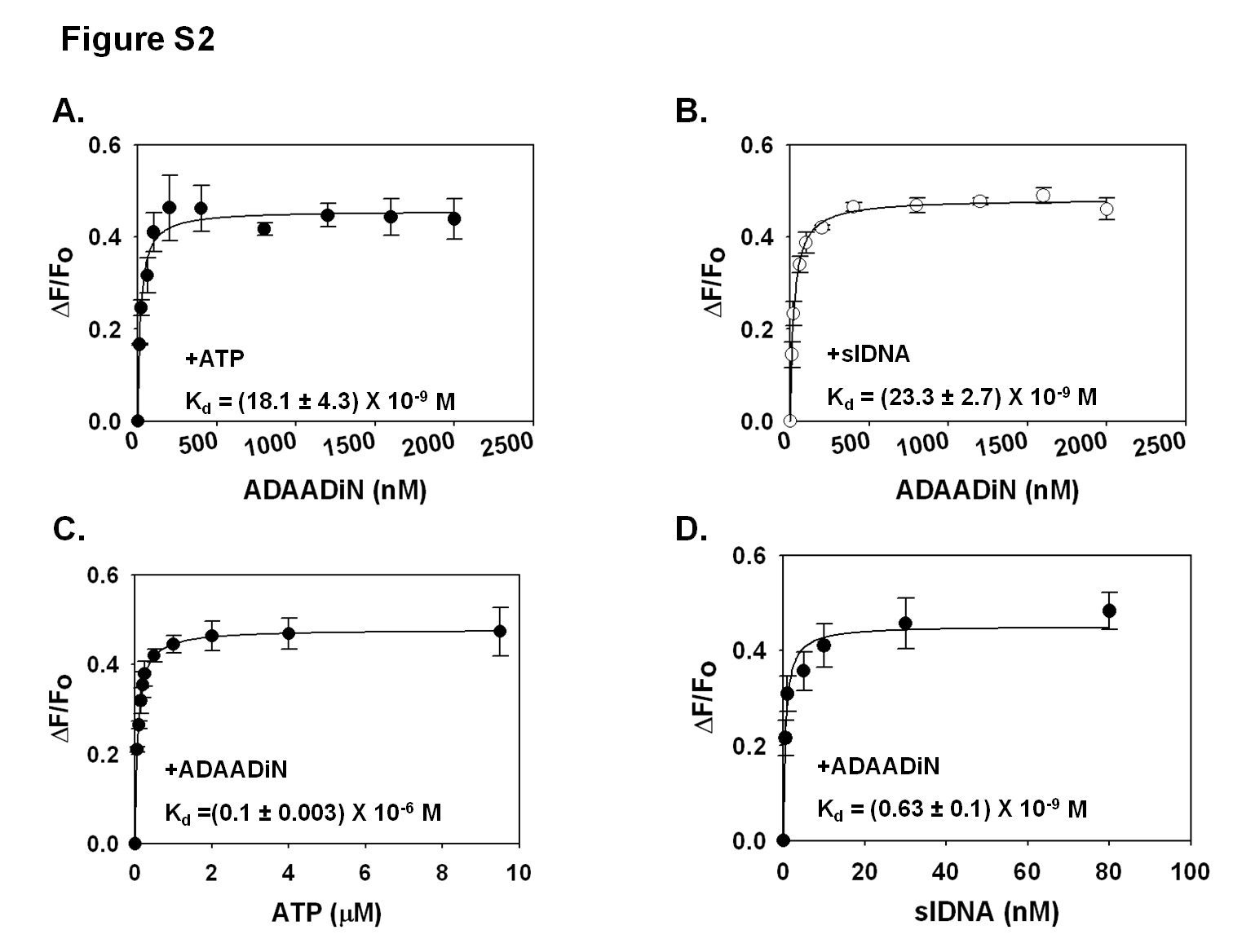

Supplement: Figure S2 — Binding of ADAADiN, ATP, and slDNA to ADAAD. (A). Binding of ADAADiN to ADAAD in the presence of 40 μM ATP. (B). Binding of ADAADiN to ADAAD in presence of 3 μM slDNA. (C). Binding of ATP in the presence of 2 μM ADAADiN. The Kd for the interaction was calculated to be 0.1±0.003 μM, suggesting that in the presence of ADAADiN, ATP binds to the protein with 10-fold higher affinity than in the absence of the inhibitor. (D). Binding of slDNA to ADAAD in the presence of 2 μM inhibitor. The Kd was calculated to be 0.63±0.1 nM, again suggesting that slDNA binds with higher affinity to the protein in the presence of ADAADiN. (TIFF) [file pone.0049822.s002.tiff]

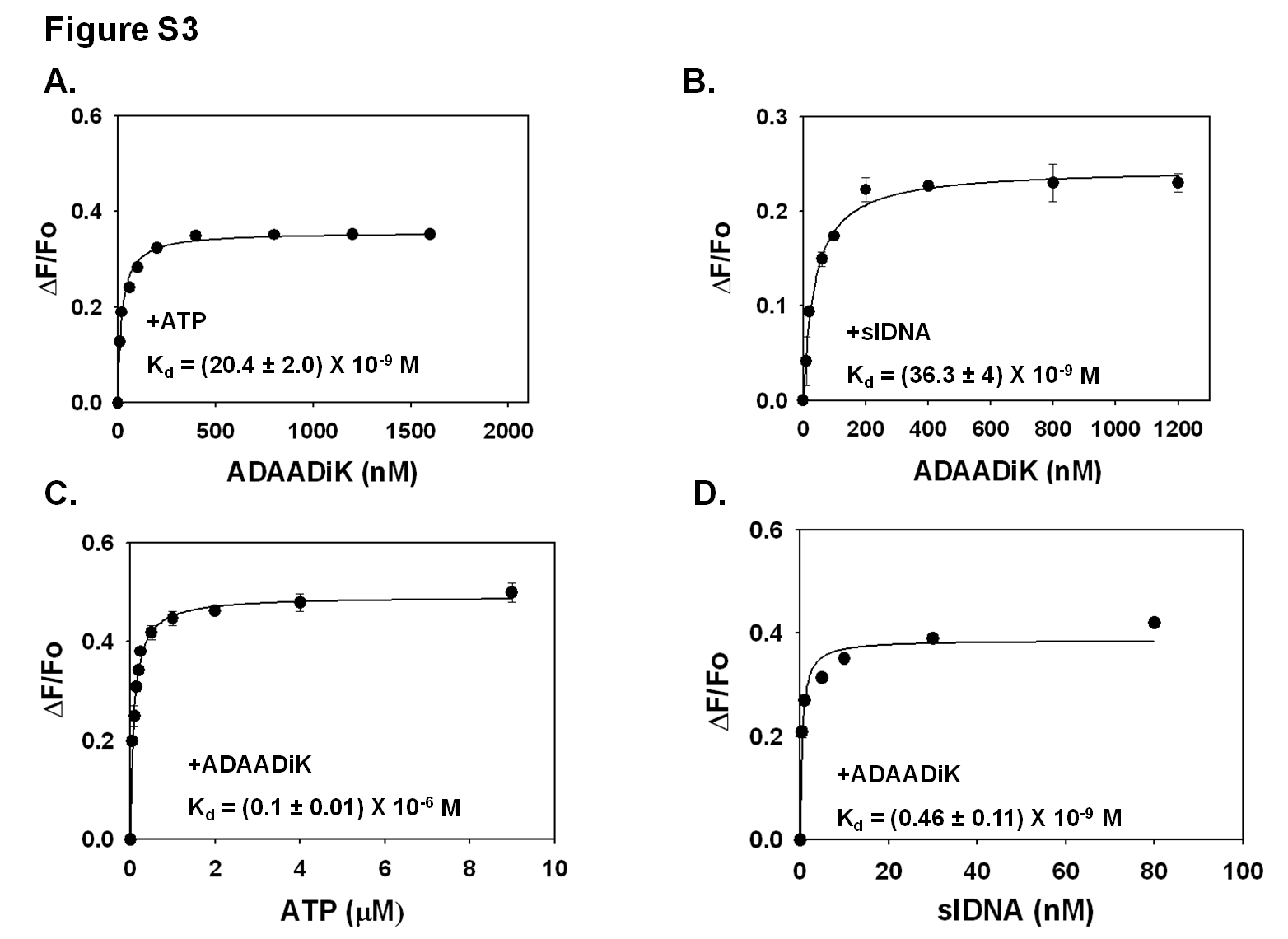

Supplement: Figure S3 — Binding of ADAADiK, ATP, and slDNA to ADAAD. (A). Binding of ADAADiK to ADAAD in the presence of 40 μM ATP. (B) Binding of ADAADiK to ADAAD in presence of 3 μM slDNA. (C). Binding of ATP in the presence of 2 μM ADAADiK. The Kd for the interaction was calculated to be 0.1±0.01 μM, suggesting that in the presence of ADAADiK, ATP binds to the protein with 10-fold higher affinity than in the absence of the inhibitor. (D). Binding of slDNA to ADAAD in the presence of 2 μM inhibitor. The Kd was calculated to be 0.46±0.11 nM, again suggesting that slDNA binds with higher affinity to the protein in the presence of ADAADiN. (TIFF) [file pone.0049822.s003.tiff]

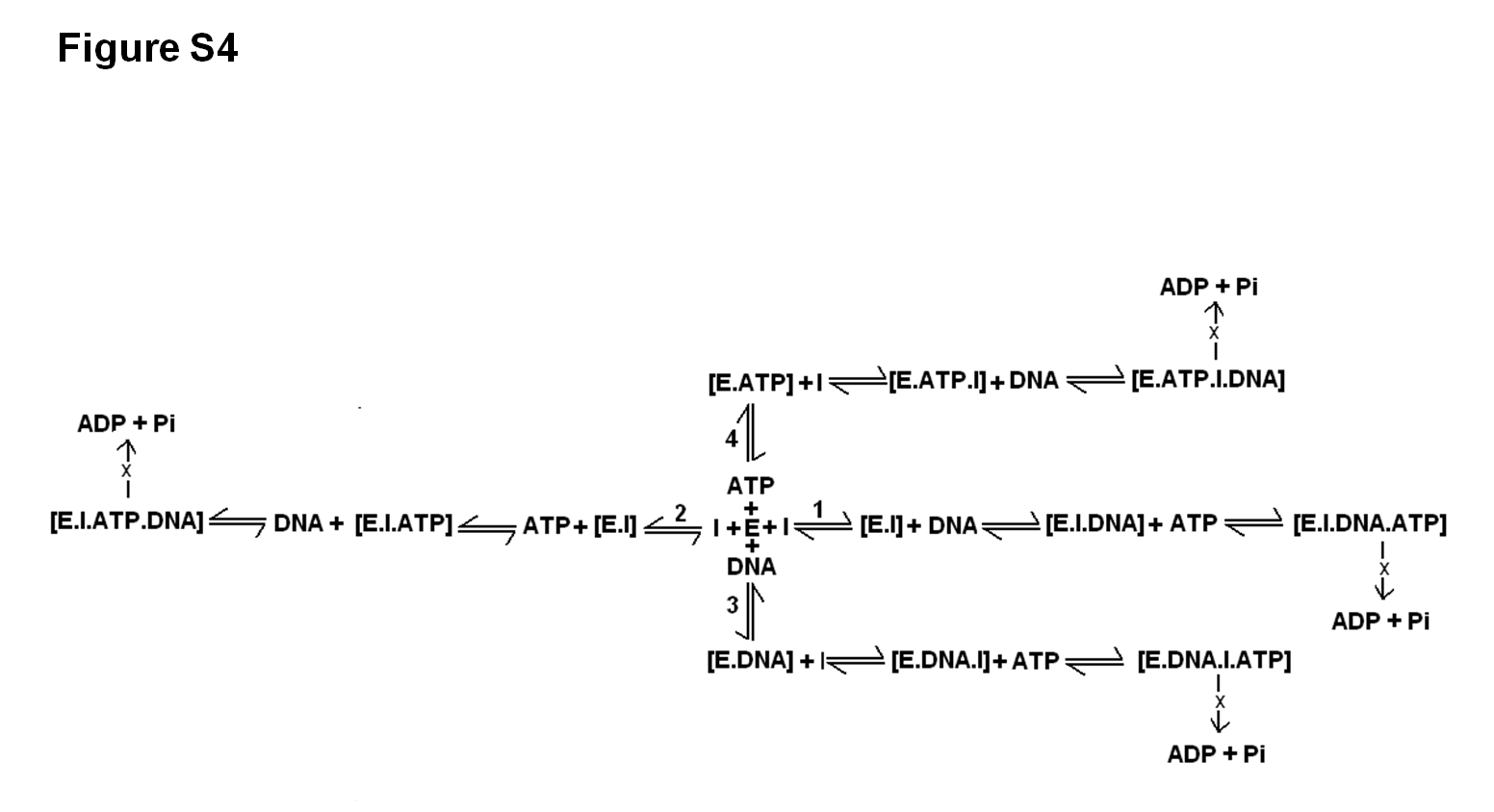

Supplement: Figure S4 — Model for the interaction of ATP, stem-loop DNA, and ADAADiN with ADAAD. ADAAD (E) can interact with ADAADi (I) in the absence of both ATP and DNA to form a binary complex [EI]. This complex can further interact either with ATP or DNA, such that these ligands bind to the protein with higher affinity. The ternary complex, [E.I.ATP] or [E.I.DNA], can subsequently interact with DNA or ATP but this interaction does not lead to ATP hydrolysis, presumably because the conformation of the complex does not allow ATP to be hydrolyzed. (TIFF) [file pone.0049822.s004.tiff]

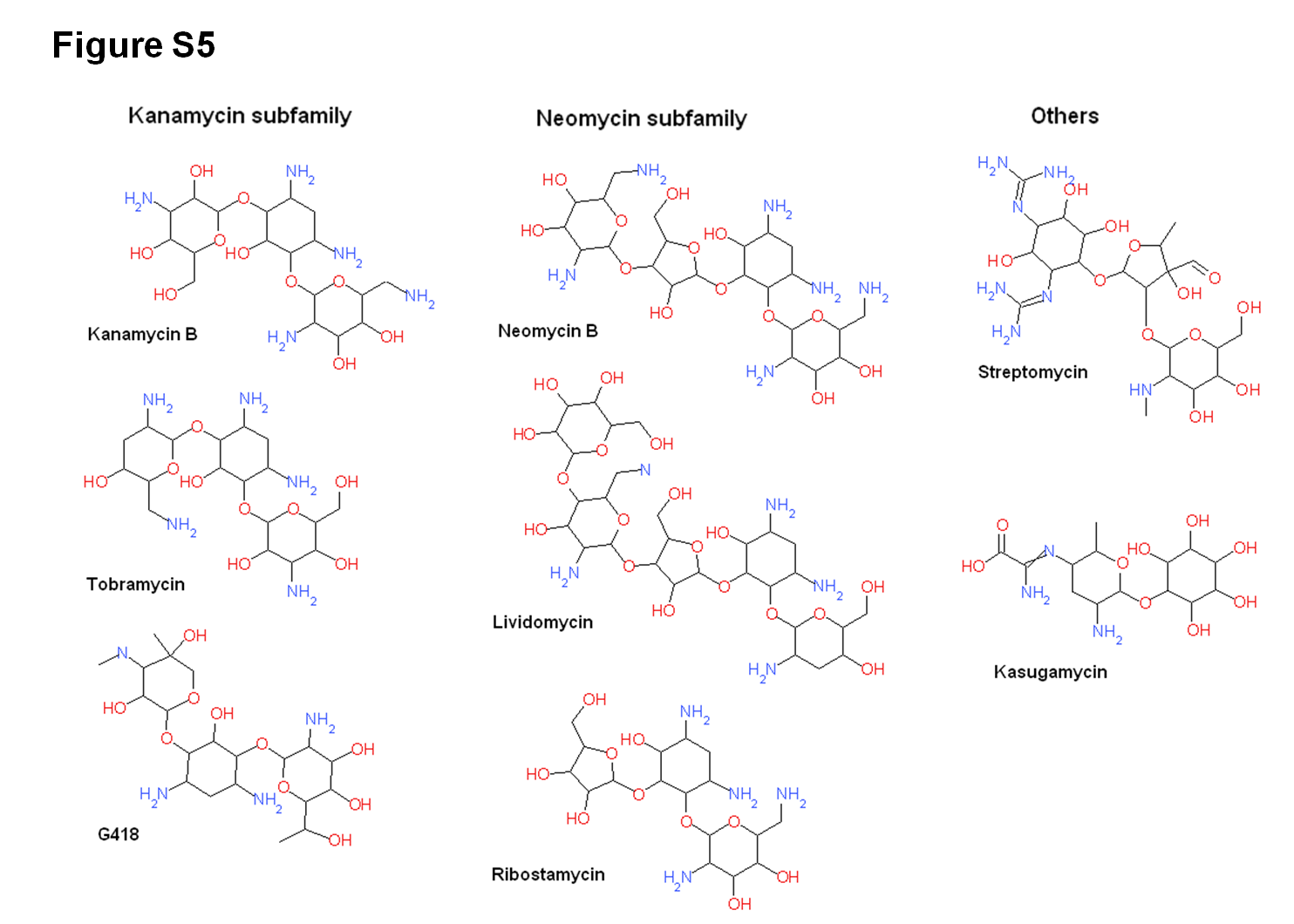

Supplement: Figure S5 — Structure of aminoglycosides. The kanamycin sub family consists of kanamycin, tobramycin, and G418. The neomycin sub-family consists of neomycin, lividomycin, and ribostamycin. Others like streptomycin and kasugamycin lack the central deoxystreptidine ring. (TIFF) [file pone.0049822.s005.tiff]

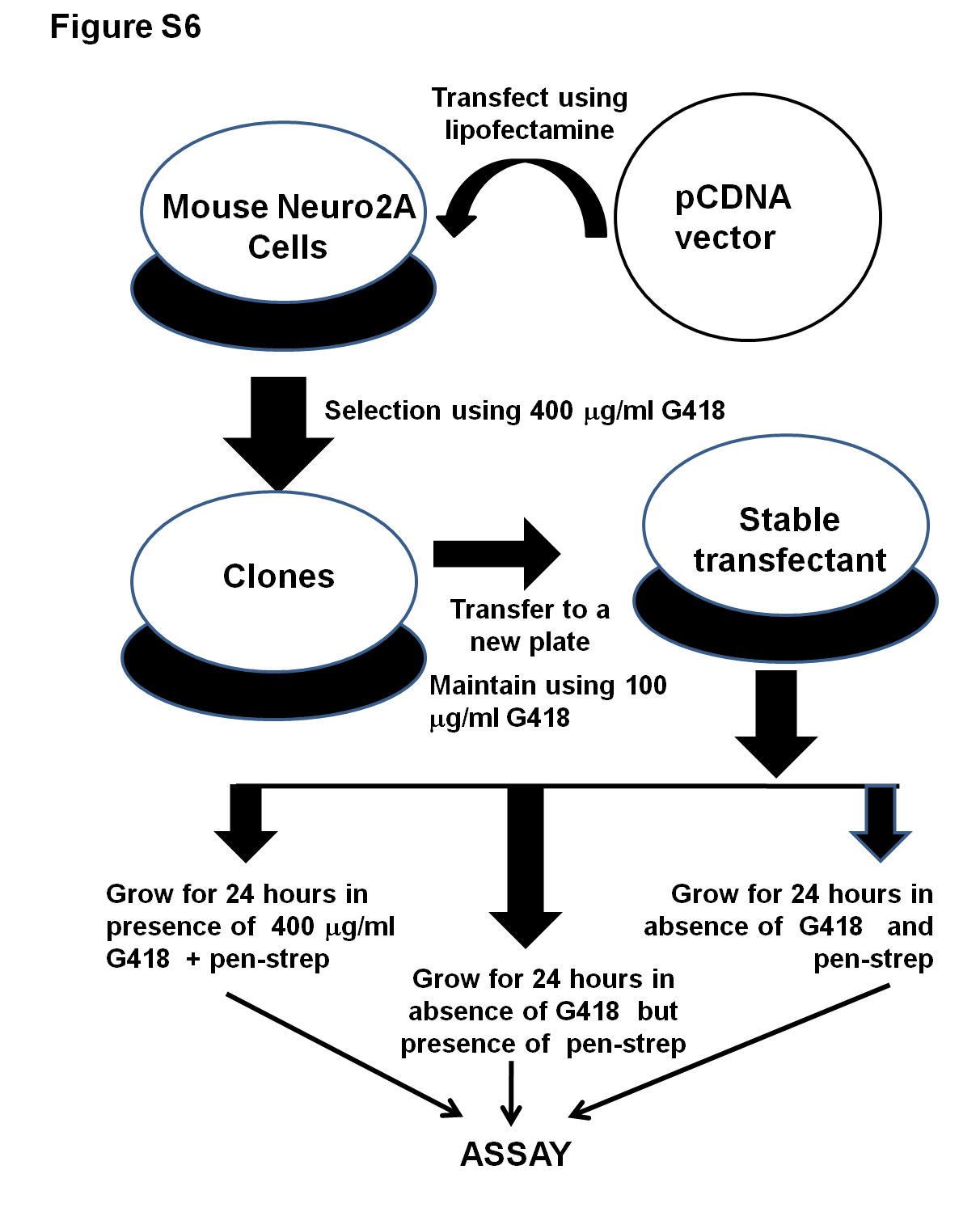

Supplement: Figure S6 — Creating stable aph transfected cell lines and assay conditions. Mouse Neuro2A cells were transfected with pcDNA 3.1 myc/his (−) using Lipofectamine (Life Technologies). After transfection cells were selected in the presence of 400 μg/ml G418 in the growth media till clones were obtained. Single clones were transferred to new plate and maintained in the presence of 100 μg/ml G418 in the growth media. For studying the effect of ADAADi produced inside the cells by the action of vector-encoded APH enzyme, cells were grown for 24 hours, prior to assay, as follows: i) in the presence of 400 μg/ml G418 and pen-strep; ii) in the absence of G418 but presence of pen-strep; iii) in the absence of both G418 and pen-strep. The same protocol was used for analyzing the expression of SG2NA variants. The only exception was that the cells were grown for 12 hours, prior to assay, as follows: i) in the presence of 400 μg/ml G418 and pen-strep; ii) in the absence of G418 but presence of pen-strep; iii) in the absence of both G418 and pen-strep. (TIFF) [file pone.0049822.s006.tiff]

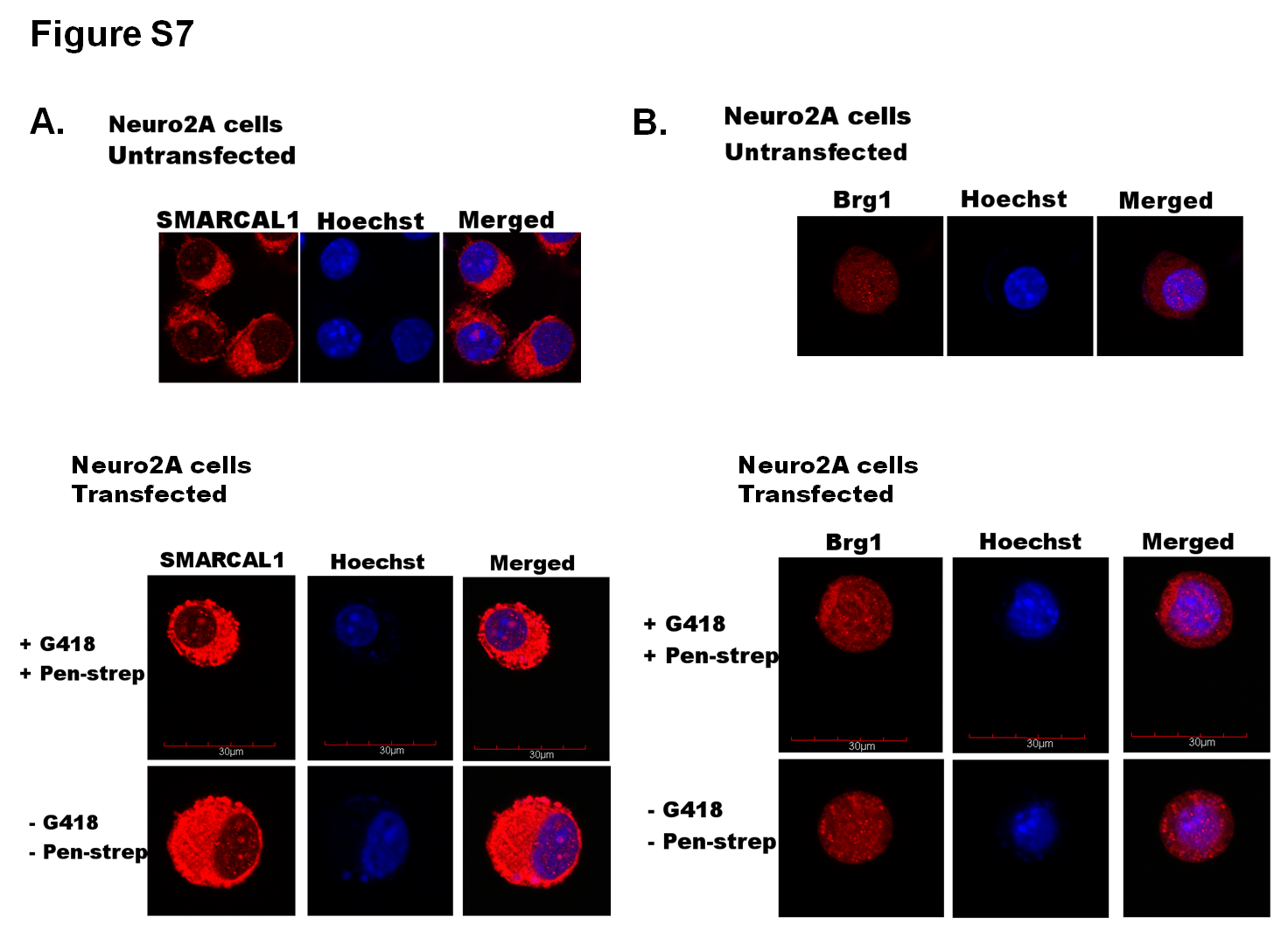

Supplement: Figure S7 — Localization of SWI2/SNF2 proteins is not altered in transfected cells. (A). Localization of SMARCAL1 in untransfected (top) and stably transfected (bottom pairs) Neuro2A cells. Following selection of a stable transfectant, the cells were grown either in the presence or absence of antibiotics and studied using polyclonal antibiodies raised against the N-terminal region of SMARCAL1. (B). Localization of Brg1 in untransfected and stably transfected Neuro2A cells grown either in the presence or absence of antibiotics was studied using monoclonal antibody against Brg1. The secondary antibody in both cases was conjugated to TRITC and the nucleus was stained using Hoechst. (TIFF) [file pone.0049822.s007.tiff]

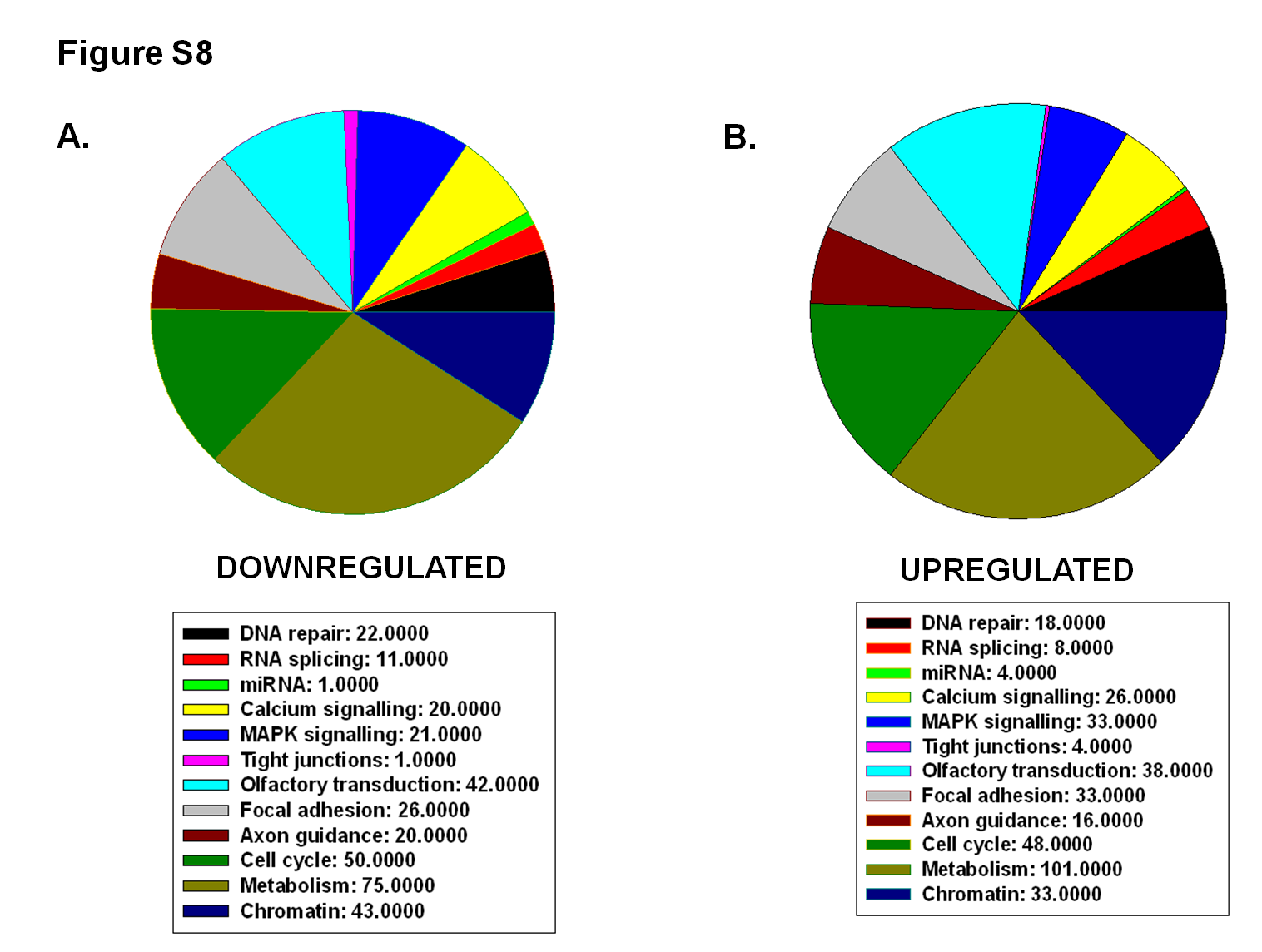

Supplement: Figure S8 — Gene expression is altered in stably transfected Neuro2A cells. The gene expression in transfected Neuro2A cells grown in the presence of antibiotics was compared with the the expression profile in untransfected Neuro2A cells. The number indicates the number of genes upregulated or downregulated. (TIFF) [file pone.0049822.s008.tiff]
