# Supplementary material for: Global Epigenetic Changes Induced by SWI2/SNF2 Inhibitors Characterize Neomycin-Resistant Mammalian Cells
Source: PLoS One. 2012 Nov 28;7(11):e49822. doi: 10.1371/journal.pone.0049822 (PMC3509132; doi:10.1371/journal.pone.0049822)
Supplement: Table S1 — List of primers used for RT-PCR and ChIP analysis. (DOC) [file pone.0049822.s009.doc]

**Table S1:** List of primers used for RT-PCR and ChIP analysis.

| **Gene** | **Used for** | **Forward primer (5'-3')** | **Reverse primer (5'-3')** |
| --- | --- | --- | --- |
| β-actin | RT-PCR | GCCTCACTGTCCACCTTCCA | GGGCCGGACTCATCGTACT |
| GAPDH | RT-PCR | GGTCGGAGTCAACGGATT | GAGGGATCTCGCTCCTGG |
| 78 kDa SG2NA | RT-PCR | GAATGGGCTGAACCAATAACG | CCGTCAAGTCTGCAAGGTCTC |
| 87 kDa SG2NA | RT-PCR | CATGGAATACCTACATCAG | TAGAAGGCACAGTCGAGG |
| Nanog | RT-PCR | AAGGCTGCGGCTCACTTCCT | TGTGGGGACCAGGAAGACCCAC |
| Dicer | RT-PCR | GGGTTCTGCATTTAGGAGCTAGATGAG | TCCACGAGTCACAATCAACACGG |
| ADH4 | RT-PCR | AATGACTATACCCACCGTGGAC | TTCCTTCCTTCATCAGGTCAAT |
| EP300 | RT-PCR | GAACAGCCCTGGATTAAGTTTG | ATGACTTGATTGGGCATTATCC |
| Runx2 | RT-PCR | GCCGGGAATGATGAGAACTA | GGTGAAACTCTTGCCTCGTC |
| SG2NA | ChIP | CAAAACACGCCAGGAATTCTA | TTTCTACGCCTGTGGAATCTG |
