# Supplementary material for: Global Epigenetic Changes Induced by SWI2/SNF2 Inhibitors Characterize Neomycin-Resistant Mammalian Cells
Source: PLoS One. 2012 Nov 28;7(11):e49822. doi: 10.1371/journal.pone.0049822 (PMC3509132; doi:10.1371/journal.pone.0049822)
Supplement: Table S2 — Primers used for amplifying aph (3′)-I and aph (3′)-IIa genes. aph (3′)-I was amplified from pET 28a (+) and aph (3′)-IIa was amplified from pCDNA 3.1 vector. (DOC) [file pone.0049822.s010.doc]

**Table S2:** Primers used for amplifying *aph (3')-I* and *aph (3')-IIa* genes. *aph (3')-I* was amplified from pET 28a (+) and *aph (3')-IIa* was amplified from pCDNA 3.1 vector.

| **Gene** | **Forward primer (5'-3')** | **Reverse primer (5'-3')** |
| --- | --- | --- |
| *aph (3')-I* | GCCATATGATGAGCCATATTCAAC GGG | GGATCCTTAGAAAAACTC ATC |
| *aph (3')-IIa* | GCTCGAGATGATTGAACAAGA | GGGATCCTCAG AGAACTCGT CAA |

**Table S3:** P-values calculated for RT-PCR data showing alteration in expression of ADH4, Nanog, RUNX2, EP400 and Dicer1.

|  | P-value  (Untransfected) | P-value  (Transfected cells  Presence of antibiotics) | P-value  (Transfected cells  Absence of antibiotics) |
| --- | --- | --- | --- |
| ADH4 | 0.0025 | 0.041 | 8.52E-05 |
| Nanog | 2.77E-07 | 0.001 | 0.5828 (Not significant at P<0.05) |
| Runx2 | 0 | 1.465E-07 | 2.62E-08 |
| EP300 | 3.74E-05 | 0.041 | 6.07E-06 |
| Dicer1 | 0 | 3.51E-05 | 6.53E-07 |
